# Water‐Assisted Programmable Assembly of Flexible and Self‐Standing Janus Membranes

**DOI:** 10.1002/advs.202305239

**Published:** 2023-10-24

**Authors:** Qun Yi, Mingyue Qiu, Xiaoyu Sun, Haonan Wu, Yi Huang, Hongxue Xu, Tielin Wang, William Nimmo, Tian Tang, Lijuan Shi, Hongbo Zeng

**Affiliations:** ^1^ School of Chemical Engineering and Pharmacy Hubei Key Lab of Novel Reactor & Green Chemical Technology Key Laboratory of Green Chemical Engineering Process of Ministry of Education Wuhan Institute of Technology No.206 Guanggu Road, East Lake New Technology Development Zone Wuhan 430072 China; ^2^ Department of Chemical and Materials Engineering University of Alberta 9211‐116 Street NW Edmonton Alberta T6G 1H9 Canada; ^3^ Energy Engineering Group Energy 2050 University of Sheffield Western Bank Sheffield S3 7RD UK; ^4^ Department of Mechanical Engineering University of Alberta 9211‐116 Street NW Edmonton Alberta T6G 1H9 Canada

**Keywords:** Janus membrane, H‐bonded assembly, unidirectional water transport, vapor response

## Abstract

Janus membranes with asymmetric wettability have been considered cutting‐edge for energy/environmental‐sustainable applications like water/fog harvester, breathable skin, and smart sensor; however, technical challenges in fabrication and accurate regulation of asymmetric wettability limit their development. Herein, by using water‐assisted hydrogen‐bonded (H‐bonded) assembly of small molecules at water/oil interface, a facile strategy is proposed for one‐step fabrication of membranes with well‐regulable asymmetric wettability. Asymmetric orderly patterns, beneficial for mass transport based on abundant high‐permeability sites and large surface area, are constructed on opposite membrane surfaces. Upon tuning water‐assisted H‐bonding via H‐sites/configuration design and temperature/pH modulation, double‐hydrophobic, double‐hydrophilic, and hydrophobic‐hydrophilic membranes are facilely fabricated. The Janus membranes show smart vapor‐responsive curling and unidirectional water transport with promising flux of 1158±25 L m^−2^ h^−1^ under natural gravity and 31500±670 L·(m^−2^ h^−1^ bar^−1^) at negative pressure. This bottom‐up approach offers a feasible‐to‐scalable avenue to precise‐manipulation of Janus membranes for advanced applications, providing an effective pathway for developing tailor‐made self‐assembled nanomaterials.

## Graphical abstract

The fabrication of flexible and self‐standing Janus membranes, which presents a significant technological challenge, is achieved through a one‐pot assembly of small molecules at the oil/water interface. The precise construction of double‐hydrophobic, double‐hydrophilic, and hydrophobic‐hydrophilic membranes with tailored wettability asymmetry is achieved through H‐site configuration, temperature, and pH regulation. The Janus membranes exhibit intelligent vapor response and rapid unidirectional water transport with a promising flux.

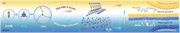

## 1 Introduction

Janus membranes possessing asymmetric wettability on opposite sides exhibit unique performances like humidity response and water unidirectional transport, thus are promising for energy/environmental‐sustainable applications such as water purification, fog collector, humidity sensor, breathable skin and smart robot.^[^ ^1^, ^2^, ^3^, ^4^, ^5^ ^]^ Till now, Janus membranes are typically mixed‐matrix membranes formed through the single‐face modification of a porous substrate or the separate fabrication and subsequent combination of two opposite layers.^[^ ^1^, ^2^, ^3^, ^4^, ^5^ ^]^ Challenges of traditional synthesis methods for Janus membranes mainly lies on two aspects: I) entire modification of the opposite sides of the porous substrates due to capillary effects; 2) limited membrane stability caused by the weak interfacial compatibility between the two layers. Moreover, the transport/response performance of Janus membranes is closely linked to the wettability differentiation on opposite sides and the surface patterns.^[^ ^2^, ^6^, ^7^, ^8^ ^]^ The regulation of the wettability asymmetricity is hard to be reached technically in traditional methods.^[^ ^2^ ^]^ Orderly patterns on the membrane surface can bring about abundant high‐permeability sites and large surface areas to accelerate the mass transport,^[^ ^6^, ^7^, ^8^ ^]^ which still faces a great difficulty since their emergence requires special thermodynamic non‐equilibrium reaction processes.^[^ ^6^ ^]^ Developing a facile‐to‐scale‐up approach to fabricate Janus membranes with orderly surface patterns and desirable asymmetric wettability is therefore of vital significance for accessing ultra‐rapid transport/response efficiency.

Interfacial synthesis strategy (such as interfacial polymerization and interfacial assembly) has been demonstrated as a powerful tool for constructing advanced functional membranes.^[^ ^9^, ^10^ ^]^ Typical interfacial polymerization occurred on the surface of a substrate,^[^ ^11^ ^]^ as a popular tool for coating a substrate to prepare a mixed‐matrix Janus membrane, often suffers from the technological challenges faced by the single‐face modification method. Alternatively, liquid‐liquid interfacial assembly strategy is of high promise to weave self‐standing single‐matrix membrane from bottom to up, thus mitigating the issue of weak interfacial compatibility observed in many traditional Janus membranes. As a typical thermodynamic non‐equilibrium process at immiscible liquid‐liquid diphase system, moreover, liquid‐liquid interfacial synthesis strategy can endow membranes with orderly surface patterns.^[^ ^6^, ^7^, ^8^, ^9^, ^10^ ^]^ Up to now, however, the typical membranes prepared through liquid‐liquid interfacial assembly are symmetric membranes, and it is still a challenge for constructing Janus membranes possessing orderly patterns based on this strategy. Inspired by the fact that different nanostructures can be accessed from the same synthon under kinetical or thermodynamical control over the assembly pathway,^[^ ^12^, ^13^, ^14^ ^]^ it's of high feasibility for constructing asymmetric membranes if we differentiate the assembly pathway in the immiscible two phases during the interfacial assembly process in the immiscible two phases to construct asymmetric membranes. Considering that the hydrogen bonding (H‐bonding) type can be differentiated in oil and water to trigger varied assembly pathway,^[^ ^15^, ^16^ ^]^ H‐bonding driven interfacial assembly is particularly credible. Especially, water can involve in H‐bonded assembly pathway as a natural H‐bond acceptor & donor,^[^ ^17^ ^]^ and the type/intensity of water‐involved H‐bonds is highly sensitive to external stimulus (e.g., pH, temperature).^[^ ^15^, ^16^ ^]^ Consequently, water‐assisted H‐bonded interfacial assembly is hopeful for constructing Janus membranes with controllable asymmetric surface pattern and wettability.

**Scheme 1:**
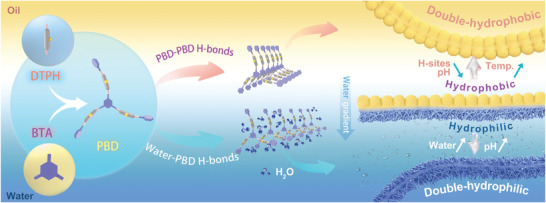
Design principle of Janus membranes. Design principle of the construction of self‐standing and flexible Janus membranes via H‐bonding assembly at oil/water interface.

Herein, we propose a H‐bonded interfacial assembly strategy in oil/water system for one‐step fabrication of membranes with regulable asymmetric surface patterns (see **Scheme** **1**). We have designed a flexible H‐sites‐functionalized small molecule (named as DTPH) to assembly with π‐conjugated linker (named as BTA) into dynamic imine polymer (named as PBD), considering that conformational flexibility allows the formation of different H‐bonding types and π‐π stacking can promote neighboring H‐bonding.^[^ ^18^ ^]^ H‐bonding can be differentiated into that between PBD molecules themselves (named as PBD‐PBD H‐bonding) in oil and that between water and PBD molecules (named as water‐PBD H‐bonding) in water, triggering the fabrication of hydrophobic nanospheres and hydrophilic nanofibers, respectively. Then, water gradient with proper thickness has been constructed at the interfacial region by regulating polarity differentiation between oil and water, finally bridging hydrophobic and hydrophilic assemblies into a self‐standing Janus membrane. Upon further switching on/off H‐bonding via H‐sites/configuration/temperature/pH regulation, the surface pattern and wettability have been controllably regulated. Unidirectional water transportability with outstanding water flux and humidity‐sensitive curling are achieved, behaving multifunctionality of humidity sensor, water/fog harvesting, breathable skin, etc.^[^ ^1^, ^2^, ^3^, ^4^, ^5^ ^]^ Moreover, this work focuses on the crucial formation mechanism for the self‐standing Janus membrane, providing a facile approach for precisely constructing membranes with desired functions.

## 2 Results and Discussion

### 2.1 One‐Pot Fabrication of Flexible and Self‐Standing Janus Membranes

**Figure 1:**
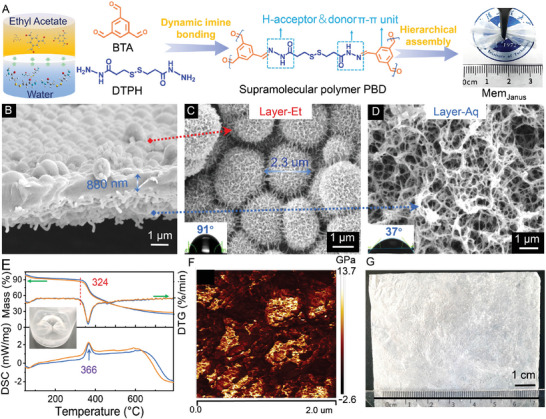
Fabrication and characterization of Janus membranes. A) Schematic diagram of interfacial self‐assembly of 1,3,5‐benzenetricarboxaldehyde (BTA) and 3,3′ – dithiobis(propionyl hydrazine (DTPH) at the ethyl acetate (EtOAc)/water interface at 40 °C. B–D) SEM images of the cross section (B), Layer‐Et (C), and Layer‐Aq (D) of the Janus membrane (Mem_Janus_); Inset: static water contact angle. E) TGA, DTG and DSC curves of Mem_Janus_ (bule lines) before and (red lines) after liquid nitrogen treatment for 24 h. Inset: photo of Mem_Janus_ in liquid nitrogen. F) Young's modulus of Mem_Janus_ detected from AFM characterization. G) Picture of large‐sized Mem_Janus_ fabricated through a scale‐up approach.

Upon the self‐assembly of a H‐sites‐functionalized small molecule 3,3′‐dithiobis(propionyl hydrazine (DTPH) and 1,3,5‐benzenetricarboxaldehyde (BTA) at the ethyl acetate (EtOAc)/water interface at 40 °C, typically, a self‐standing and flexible Janus membrane (denoted as Mem_Janus_) composited of interconnected urchin‐like microspheres on the EtOAc side (named as Layer‐Et) and nanofiber networks on the water side (named as Layer‐Aq) is fabricated (**Figure** **1**A–D). Contact angle measurement shows that the Layer‐Et is hydrophobic with a static water contact angle of 91°, while the Layer‐Aq is hydrophilic with a static water contact angle of 37°. Thermogravimetric analysis (TGA) and differential scanning calorimetry (DSC) characterizations show that the Mem_Janus_ has a high thermostability with an initial decomposition temperature of 324 °C (Figure 1E). It is noted that the Mem_Janus_ remains stable after liquid nitrogen treatment for 24 h (inset in Figure 1E), demonstrating the as‐prepared membrane possesses good endurance for both high‐temperature and low‐temperature. The average Young's modulus of the Mem_Janus_ determined using an atomic force microscope (AFM) is ≈1.8 GPa (Figure 1F). The tensile stress‐strain test of the Mem_Janus_ reveals that the maximum tensile strength of the membrane is ≈26 MPa and the Young's modulus calculated from the ratio of stress to strain is 1.1 GPa (Figure S1, Table S1, Supporting Information). The result reveals a promising mechanical property of the Mem_Janus_ compared with conventional polymer materials (Table S1, Supporting Information).^[^ ^19^, ^20^, ^21^, ^22^ ^]^ It is known that the scale‐up ability is a key indicator for a real application of membrane materials. On the basis of the mild preparation condition, easily obtainable building blocks and commercial solvents, the Mem_Janus_ can be facilely enlarged on a large‐sized oil/water interface (Figure 1G, scanning electron micrograph (SEM) image see Figure S2A, Supporting Information). It is noted that without the support of substrates, the stability and integrity of self‐standing Janus membrane during scale up should be highly focused. Benefiting from the relatively high bond energy, directionality unique and reversible nature of dynamic covalent bonding and hydrogen bonding, the assembly process can continuously undergo “self‐reading” and “self‐correcting”,^[^ ^15^, ^16^, ^17^, ^18^ ^]^ thus ensuring the stability and integrity of self‐standing Janus membrane. The average Young's modulus of the large‐scale Mem_Janus_ after being stored under room condition for eight months can reach about 1.7 GPa (Figure S2B, Supporting Information), consistent well with that of the freshly prepared sample (1.8 GPa, Figure 1F).

It is noted that the Janus structure can be quickly constructed within a few minutes (Figure S3, Supporting Information). On the Layer‐Et, smooth nanospheres are interconnected into urchin‐like microspheres as assembly time goes by (Figure S3E–H, Supporting Information). Meanwhile, the evolution from nanospheres into short nanofibers and then into fibric networks occurs on the Layer‐Aq (Figure S3I–L, Supporting Information). By controlling the assembly time, the thickness of the membrane can be tuned from ca. 280 to ca. 1.1 um (Figure 1B; Figure S3A–D, Supporting Information). The elements and their chemical states in different regions of the Mem_Janus_ (i.e., Layer‐Et, Layer‐Aq and interlayer) have been investigated by using X‐ray photoelectron spectroscopy (XPS) etched for different depth. O, N, C, and S elements with almost constant content can be observed throughout the membrane (Figure S4, Supporting Information). Fourier transform infrared (FT‐IR) and solid ^13^C nuclear magnetic resonance (NMR) spectra of the Mem_Janus_ further prove that DTPH and BTA are assembled into dynamic covalent polymer PBD though dynamic imine bonding between amino group and aldehyde group (Figure 1A, detailed analysis see Figure S5, Supporting Information).^[^ ^23^ ^]^ It can be demonstrated from the above analysis that the formation of the Janus structure is kinetically‐dependent on the hierarchical self‐assembly of the dynamic covalent polymer PBD.

### 2.2 Formation Mechanism of the Janus Structure and Self‐Standing Nature

**Figure 2:**
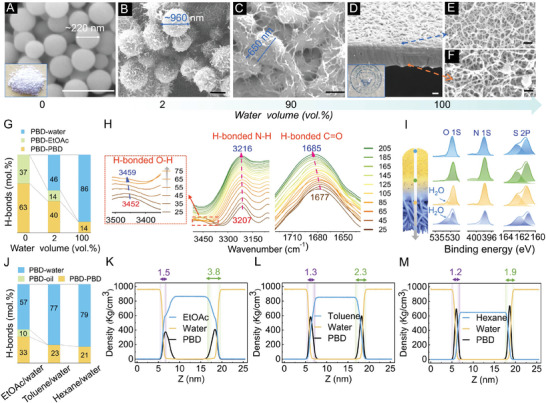
Driving force for the formation of the Janus structure and self‐standing nature. A–F) SEM images of the product via the self‐assembly of BTA and DTPH in EtOAc solution with water addition; water volume (vol. %): 0 (A), 2 (B), 90 (C) and 100 (D–F); Scale bars: 500 nm. G) MD simulated proportion of PBD‐involved H‐bonds in EtOAc, water (2 vol%)/EtOAc mixture and water. H) FT‐IR spectra of the Mem_Janus_ obtained at different temperature conditions (25 to 215 °C) in the ν_O‐H_, ν_N‐H_ and ν_C = O_ regions. I) XPS depth profiling down to 150 nm for the Mem_Janus_ formed at 30 min. J) MD simulation calculated proportion of PBD‐involved H‐bonds in different systems. K–M) Density profiles of organic solvents, water and PBD averaged over the last 10 ns for EtOAc/water interface (K), toluene/water interface (L) and hexane/water interface (M); Purple and green ranges represent the thickness of water gradient and interfacial region, respectively.

The driving force for the differentiation of the architectures in oil and water has been investigated. The assembly performances of PBD molecules in bulk EtOAc, EtOAc/water mixed system and bulk water have been compared experimentally and computationally. In pure EtOAc, powders comprised of smooth nanospheres with diameters of ca. 220 nm are formed (**Figure** **2**A). As trace water (2 vol.%) is added to EtOAc, nanospheres gradually grow up with “thorns” on the surface (Figure 2B). In the EtOAc/water mixed solution containing large content of water (90 vol.%), nanospheres are highly fused accompanied by the growth of nanofibers on the surface (Figure 2C). In pure water, as expected, fibric networks are constructed to form transparent membrane (Figure 2D–F).

The formation mechanism of nanospheres in oil has been investigated. Variable‐temperature FT‐IR spectra of the formed nanospheres have been detected (see Figure S6, Supporting Information). The characteristic signals of intermolecular H‐bonded ν_N‐H_ at 3207 cm^−1^ (no free ones with the frequency higher than 3400 cm^−1^) and H‐bonded ν_C = O_ at 1677 cm^−1^ shift to high frequency accompanied by weakened peak intensity with increasing temperature, demonstrating the presence of C = O···N‐H typed H‐bonding between PBD molecules themselves (named as PBD‐PBD H‐bonding).^[^ ^15^ ^]^ Molecular dynamics (MD) simulations about the relations between PBD assemblies and the contribution of H‐bonds between different groups have been performed (simulation details see Supporting Information), aiming to provide mechanistic understanding on the assembly performance in different environments. Considering the large size of PBD polymer, a small unrepeatable unit from the polymer structure was regarded as the computational model (Figure S7, Supporting Information). It has been confirmed that the number of PBD‐PBD H‐bonds is much higher than that of H‐bonds between PBD and EtOAc (named as PBD‐EtOAc H‐bonds) (Figure 2G). As a contrast, diaminooctane (DAO) that has no H‐bond sites has been employed to replace DTPH to assembly with BTA at the ethyl acetate (EtOAc)/water interface at 40 °C. It has been observed that only irregular assemblies are formed (Figure S8, Supporting Information). According to the above results, it can be proven that the PBD‐PBD H‐bonding plays a crucial role in the assembly of nanospheres in pure EtOAc.

To further illustrate the evolution mechanism from nanospheres to nanofibers with water addition, XPS depth profiling and variable‐temperature FT‐IR spectroscopy of the Mem_Janus_ have been detected. H‐bonded ν_O‐H_ on water (3450 cm^−1^), ν_N‐H_ (3207 cm^−1^) and ν_C = O_ (1677 cm^−1^) are present in low temperature regime (25–75 °C) of the variable‐temperature FT‐IR spectra (Figure 2H). All the above peaks shift remarkably to high frequency followed with decreased intensity as temperature increases, proving the presence of water‐PBD H‐bonding.^[^ ^14^ ^]^ The XPS depth profiling of Mem_Janus_ reveals that the characteristic O 1s signal of water molecule at 531.1 eV appears in the Layer‐Aq (Figure 2I),^[^ ^24^ ^]^ further indicating that water is involved in the fabrication of nanofibers. MD simulations of PBD molecules in bulk EtOAc, water (2 vol.%)/EtOAc mixed system and bulk water have been further performed (Figure S8, Supporting Information). It is noted that the final configurations from the simulations represent the stable equilibrated state of the ideal systems due to the difference in the scale between the realistic and simulated systems, which are not aimed to capture the kinetics of the assembly process and the real assembly arrangement. PBD molecules tend to assembly into loose arched aggregate in bulk EtOAc (Figure S10A–C, Supporting Information), and prefer to form 1D aggregate in water (Figure S9D, Supporting Information), consistent with the formation of nanofibric networks. It is noted that water molecules involve in the formation of H‐bonds with PBD molecules in the one‐dimensional aggregate (Figure S9D, Supporting Information). Meanwhile, the number of water‐PBD H‐bonds is increased significantly with water addition, which is 4 times that of PBD‐PBD H‐bonds in pure water (Figure 2G; Figure S11, Supporting Information). Combining the MD simulations and experimental characterizations, it can be proven that the transformation from PBD‐PBD H‐bonding into water‐PBD H‐bonding plays a crucial role in the evolution from nanospheres to nanofibers.

The mechanism of how the nanosphere Layer and nanofibers are connected into a self‐standing Janus membrane has been investigated. Three kinds of oil phases—EtOAc, toluene and hexane with gradually decreased polarity (4.30 vs 2.40 vs 0.06) have been employed. It has been observed that the thickness of the Mem_Janus_ turns thinner gradually with decreased polarity of oil phase (1.1 um at EtOAc/water interface vs 660 nm at toluene/water interface, see Figure S12A–C, Supporting Information). Janus membrane was failed to be formed at the hexane/water interface. Instead, separate thin membranes composited of pure nanospheres or nanofibers without connection was formed (Figure S12D, Supporting Information). That is, the formation of self‐standing Janus membrane seems related to the polarity differentiation between oil and water. MD simulation of PBD molecules at the EtOAc/water interface has been further performed. As expected, PBD molecules form great numbers of H‐bonds with water, which would disrupt the PBD‐EtOAc H‐bonds and PBD‐PBD H‐bonds (Figure 2J; Figure S13, Supporting Information). It is known that a gradient of water density is present from the EtOAc side to water side at the interfacial region (Figure S14, Supporting Information),^[^ ^25^ ^]^ which would induce an increase of water‐PBD H‐bonds from the EtOAc side to water side. In terms of the crucial role of water‐PBD H‐bonding in the evolution from nanospheres to nanofibers, it can be inferred that the water gradient from oil side to water side is responsible for the transformation from nanospheres to nanofibers.

As is known, the thickness of the interfacial region (defined as that both water and oil have >99% mass ratio) of oil‐water biphase decreases with decreased polarity of oil phase.^[^ ^26^ ^]^ As expected, the interfacial region with the presence of PBD molecules turns narrower from 3.8 to 2.3 nm and then to 1.9 nm with decreased polarity of oil phase (Figure 2K–M). Consequently, the distance for the evolution from PBD‐PBD H‐bonding to PBD‐water H‐bonding is gradually compressed, which is adverse to supporting the connection between nanospheres and nanofibers. In light of the results above, a sufficiently wide interfacial region should be established to afford a self‐standing Janus membrane.

According to the results above, it can be demonstrated that hydrophobic nanospheres formed in oil phase are gradually evolved to urchin‐like microspheres and then to hydrophilic nanofibers under the effect of water gradient from oil phase to water phase, finally inducing the in‐situ formation of self‐standing Janus membrane at the oil/water interface. As is known, traditional Janus membranes are typically mixed‐matrix membranes formed through the single‐face modification of a porous substrate or the separate fabrication and subsequent combination of two opposite layers (Scheme S1)^[^ ^2^, ^3^ ^]^ Consequently, there exists a noticeable interface between the two matrices of traditional Janus membranes, and the strength of interfacial bonding is a crucial factor affecting the stability of these Janus membranes. In sharp contrast to traditional Janus membranes, our work presents a self‐standing Janus membrane composed of a single matrix which doesn't show a pronounced interface between the two opposite layers. This unique characteristic effectively mitigates the issue of weak interfacial compatibility observed in many traditional Janus membranes. Considering the intermolecular H‐bonds between PBD molecules themselves and between PBD and water are the driving force for the formation of the Janus structure, the bonding strength has been detected experimentally and and computationally. The bonding strength of the Layer‐Et of one Janus membrane and the Layer‐Aq of another membrane was detected by using lap shear test (see Figure S15A, Supporting Information). Before test, the Layer‐Et of one Janus membrane and the Layer‐Aq of another membrane was stacked together in a moisture atmosphere for 12 h to facilitate the formation of H‐bonds. As shown in the lap shear strength curve (Figure S15B, Supporting Information), the adhesion strength between the two separate membranes is ≈23.3 kPa under the effect of intermolecular H‐bonding. Considering the maximum tensile stress of the membrane (26 Mpa) is much higher than 23.3 kPa (Figure S1, Supporting Information), the intermolecular bonding strength inside a Janus membrane is strong enough for a promising mechanical property. The time evolution of change interaction energies (ΔE) between different components in the system with PBD at water/EtOAc interface was further calculated by using MD simulation. At the final stage of simulation, as shown in Figure S16 (Supporting Information), the ΔE values for PBD‐PBD and PBD‐water were negative with much higher absolute values than those for PBD‐water, PBD‐EtOAc, and water‐EtOAc, beneficial for a high stability of the Janus membrane.

### 2.3 Modulation of the Asymmetry of Janus Membranes

**Figure 3:**
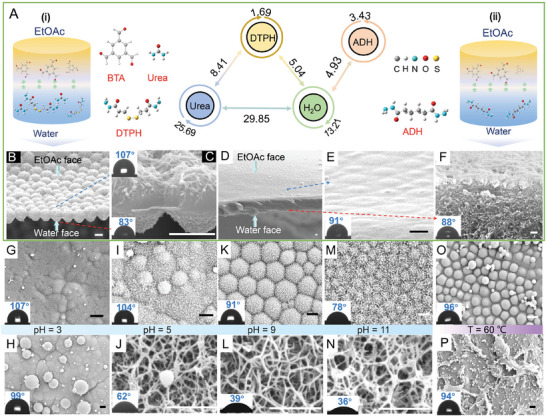
Modulation of the asymmetric property of the Janus Membranes via structural design and pH/temperature regulation. A) Schematic diagrams of the self‐assembly of BTA, urea and DTPH i) and the self‐assembly of adipic dihydrazide (ADH) and BTA ii) at the EtOAc/water interface and corresponding average hydrogen bond energies (in KJ mol^−1^). SEM images of the membrane via the self‐assembly of BTA, urea and DTPH B,C) and the membrane via the self‐assembly of adipic dihydrazide (ADH) and BTA D–F). Concentrations of BTA, urea, DTPH and ADH are 0.05, 0.75, 0.075, and 0.075 M, respectively. G–P) SEM images of the Layer‐Et (upper) and Layer‐Aq (down) of the membrane via the self‐assembly of BTA and DTPH under different temperature and pH conditions. (G,H) pH = 3; (I,J) pH = 5; (K,L) pH = 9; (M,N) pH = 11; (O,P) T = 60 °C. Scale bars: 1 um.

Given the fact that the transformation between intermolecular H‐bonding and water‐involved H‐bonding differentiates the assemblies on the two sides, we have attempted to tune the H‐bonding type to modulate the asymmetric property of the Mem_Janus_. Urea has been proven to interact strongly with kinds of H‐accepters & donors (e.g., amino acid, protein, water) through H‐bonding, and have great potential to break the intrinsic intermolecular H‐bonding between the H‐accepters & donors themselves.^[^ ^27^, ^28^ ^]^ Thus, we have tried to switch off DTPH‐water H‐bonding by employing urea as a competing H‐donor & accepter. MD simulations for DTPH in water without and with the presence of urea have been performed (details see Supporting Information). As shown in Figure S17C (Supporting Information), the DTPH‐water H‐bonds and DTPH‐DTPH H‐bonds are formed with a ratio of 565 : 342 in a system of 360 DTPH molecules in bulk water. When 900 urea molecules are added, the number of DTPH‐water H‐bonds is reduced from 565 to 509 and that of DTPH‐DTPH H‐bonds is slightly reduced from 342 to 318. Considering there exists competition between DTPH‐urea and DTPH‐water interactions, the radial distributing function (RDF) curves between DTPH and urea as well as between DTPH and water have been calculated. As shown in RDF curves in Figure S18A (Supporting Information), the two peaks in the range of 0.2–0.4 nm indicate there exits strong O···N‐H typed H‐bonds between DTPH and urea.^[^ ^27^ ^]^ In the RDF curve of DTPH and water (Figure S18B, Supporting Information), the peaks are relatively weak due to the large amount of bulk water, and the two peaks at 0.2 and 0.28 nm are relevant to the DTPH‐water H‐bonds.^[^ ^28^ ^]^ It is clear that the intensity of the two peaks drops with the addition of urea, which indicates that a lot of water molecules have been pushed away from DTPH and replaced by urea molecules.^[^ ^27^, ^28^ ^]^ DFT calculation can also prove that the intermolecular H‐bonding between DTPH and urea is much stronger than that between DTPH and water (8.41 kJ mol^−1^ vs 5.04 kJ mol^−1^, see **Figure** **3**A). Combining the RDF data with DFT calculation, it's of high feasibility for reducing the DTPH‐water H‐bonding with the addition of urea. Instead of the Mem_Janus_ comprised of nanofibers and nanospheres, as expected, a membrane comprised of orderly‐arranged uniform‐size nanospheres on both sides is fabricated (Figure 3B,C). Furthermore, the water/EtOAc system kept transparent after 48 h when the concentration of urea was increased to a hundred times that of DTPH. The failed production of membranes is mainly due to that both the DTPH‐water H‐bonds and DTPH‐DTPH H‐bonds are disrupted with the presence of urea.

Disulfide bond possesses special dihedral angle, endowing the molecules containing disulfide bonds with folded configurations.^[^ ^29^ ^]^ The binding mode of the neighboring H‐bonding sites can be altered when disulfide bond is removed.^[^ ^30^ ^]^ In this work, the folded configuration of DTPH molecule enables the H‐bonding sites on both sides of disulfide bond close to each other to interact strongly with water through C = O···H‐O‐H and N‐H···O‐H typed H‐bonding (Figure S19B,C, Supporting Information). Then, the disulfide bond on DTPH has been removed to yield adipic dihydrazide (ADH), by which the C = O···H‐O‐H and C = O···N‐H typed DTPH‐water H‐bonds have been weakened (Figure 3A; Figure S20, Supporting Information). Meanwhile, the ADH‐ADH H‐bonding is much stronger than DTPH‐DTPH H‐bonding (3.43 KJ mol^−1^ vs 1.69 KJ mol^−1^), which is also adverse to the evolution from intermolecular H‐bonding between the dynamic imine polymer to that between water and dynamic imine polymer. Correspondingly, spongy structure rather than nanofibers is fabricated on Layer‐Aq (Figure 3D–F; Figure S21, Supporting Information).

Considering that the H‐bonding type is sensitive to temperature and pH,^[^ ^15^, ^16^ ^]^ the assembly pathway has further been controlled via regulating the pH and temperature. In strongly acidic aqueous solution, the terminal amino group on DTPH is protonated in water, which is detrimental to dynamic imine bonding for the formation of dynamic imine polymer PBD.^[^ ^31^ ^]^ Moreover, not only the amino/imine groups but also water molecules prefer to be protonated under acidic condition. When reducing pH with the addition of hydrochloric acid, moreover, a large number of Cl^−^ ions exist in the solution. DFT calculation demonstrated that the H‐bonding energy between protonated DTPH and Cl^−^ is negative with a higher absolute value than that between protonated DTPH and H_2_O (−81.94 vs −70.42 kJ mol^−1^, Figure S22, Supporting Information),^[^ ^32^ ^]^ indicating the H‐bonding between protonated DTPH and H_2_O is blocked due to the presence of Cl^−^. Additionally, there aren't enough water molecules to form H‐bonds with DTPH under acidic condition due to the protonation of water molecules. As a result, the formation of nanofibers is unfavorable on the water side in the water/EtOAc diphase system at pH = 3. On the EtOAc side, meanwhile, the PBD‐PBD H‐bonding is hardly affected with pH variation due to the poor solubility of protonated DTPH and water molecules in EtOAc. Consequently, nanospheres can be constructed on the EtOAc side at pH = 3. Therefore, membrane comprised of nanospheres on both sides is fabricated at pH = 3 (Figure 3G,H). Increasing pH would facilitate the deprotonation of amino/imine groups and water molecules, which is beneficial for the formation of PBD‐water H‐bonding. Correspondingly, nanofibers are formed on Layer‐Aq (Figure 3H,J,L,N). On Layer‐Et, regular urchin‐like nanospheres are gradually formed (Figure 3G,I) and then highly fused into fabric networks (Figure 3K). The structural stability of the membranes formed at different pH conditions (i.e., pH = 3, 5, 9, 11) has been focused on. It can be seen from the FT‐IR spectra of the Janus membranes prepared at different pH that the main characteristic FT‐IR adsorption peaks are maintained well (Figure S23A, Supporting Information). Noteworthily, the signal of sulfhydryl group (2850 cm^−1^) was failed to be observed in the FT‐IR spectra, demonstrating the stability of disulfide bond of the membranes in acidic and basic conditions. The FT‐IR spectra of the Janus membranes with the soaking of HCl solution at different pH (i.e., pH = 1, 3, 5) also maintain the main characteristic signals of PBD with the absence of sulfhydryl group (Figure S23B, Supporting Information). One can see that the Janus membranes behave good acidic and basic tolerance, which is beneficial for their real application. Moreover, increasing the assembly temperature is adverse for H‐bonding between PBD and water,^[^ ^15^ ^]^ thus inducing the destruction of nanofibers in water (Figure 3P).

The surface wettability of the membranes formed with the regulation of water‐PBD H‐bonding has then been measured by using water contact angle characterization. Upon switching off water‐PBD H‐bonding by adding urea, the contact angle of Layer‐Aq and Layer‐Et is 83° and 107°, respectively (see the inset in Figure 3F). With urea participation, noteworthily, the water contact angle of Layer‐Aq is highly enhanced from 37° to 83°. Upon increasing pH, an evolution from double‐hydrophobic membrane to hydrophilic‐hydrophobic membrane and then to double‐hydrophilic membrane occurs. For the hydrophilic surface, the water contact angle is decreased in the order of Layer‐Et (pH = 11) > Layer‐Aq (pH = 5) > Layer‐Aq (pH = 9) > Layer‐Aq (pH = 3) (Figure 3G,I,K,M). The contact angle of the hydrophobic surface decreases in the order of Layer‐Et (pH = 3) > Layer‐Et (pH = 5) > Layer‐Et (pH = 9) > Layer‐Et (pH = 11) (Figure 3H,J,L,N). When temperature is increased from 40 to 60 °C, the hydrophilic‐hydrophobic membrane turns to double‐hydrophobic membrane (Figure 3O,P).

Generally, the water contact angle of a surface is mainly affected by the surface roughness, polar group and porosity.^[^ ^33^, ^34^ ^]^ To understand the crucial influencing factors of the wettability of the Mem_Janus_, we have detected both the surface roughness and surface composition of the two sides of the Janus membranes prepared under different pH conditions. First, the roughness of the two sides of the membranes was measured via AFM. As shown in Figure S24 (Supporting Information), the surface roughness factor (r, also called as the roughness ratio, *i. e*., the ratio of actual area and projection area of the surface) of the hydrophilic surface is decreased in the order of Layer‐Et (pH = 3) > Layer‐Aq (pH = 9) > Layer‐Aq (pH = 5) > Layer‐Aq (pH = 11), totally opposite to the change tendency of water contact angle. The r of the hydrophobic surface decreases in the order of Layer‐Et (pH = 3) > Layer‐Et (pH = 5) > Layer‐Et (pH = 9), which is finely consistent with the change tendency of contact angle. This result verifies that higher surface roughness contributes to higher hydrophobicity of hydrophobic surface and higher hydrophilicity of hydrophilic surface, following the Wenzel model.^[^ ^34^ ^]^ However, the water contact angle of Layer‐Aq (pH = 3) is larger than that of Layer‐Et (pH = 9), although the r of former one (r = 1.36) is smaller than that of latter one (r = 1.50). To further understand the influencing factors of contact angle, we have detected the elemental composition of the two surfaces via XPS (Table S2 and Figure S25, Supporting Information). It can be seen that the molar content of DTPH on Layer‐Aq gradually increases with increasing pH. This result is mainly because the ‐NH_2_ group on DTPH tends to be protonated under acidic conditions, which is adverse to the dynamic imine reaction of DTPH with BTA.^[^ ^31^ ^]^ Considering the polar groups (e.g., ‐NH‐, ‐S‐S‐) are originated from DTPH, the content of polar groups on Layer‐Aq is gradually increased with increasing pH, which is beneficial for enhancing the hydrophilicity of Layer‐Aq. Meanwhile, the DTPH content on Layer‐Et is slightly increased with increasing the pH, resulting in decreased hydrophobicity of Layer‐Et. The tendency above is consistent with the change of contact angle with increased pH. Moreover, water molecules are adsorbed on Layer‐Aq driven by H‐bonds (see the O 1s XPS spectra in Figure 2I), which can also increase the hydrophilicity of Layer‐Aq. Overall, both the surface roughness and polarity (or surface chemistry) are indispensable influencing factors of the surface wettability of the Janus membranes.

### 2.4 Unidirectional Vapor Response and Water Flux Performances

**Figure 4:**
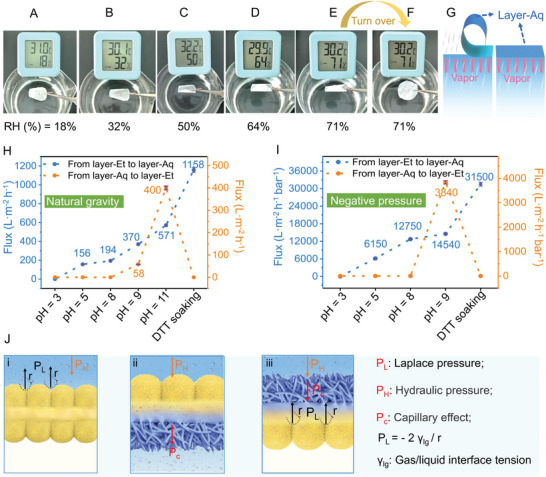
Unidirectional vapor response and water flux performances. A–F) Pictures of the selective vapor responsiveness of the Janus membrane at different humidities; Downside of the membrane is Layer‐Aq in (A–E) and Layer‐Et in (F). G) Schematic diagram of the selective vapor responsiveness of the Janus membrane; Downside of the membrane is Layer‐Aq in left graph and Layer‐Et in right graph. H,I) Water flux from layer‐Et to layer‐Aq (blue dot) and from layer‐Aq to layer‐Et (red dot) of the membranes prepared under different pH values or treated by soaking in dithiothreitol (DTT) solution at 40 °C under natural gravity (H) and negative pressure (0.4 bar) (I); The water flux is the average value of three measurements and the error bar is the standard deviation (purple line on each dot). J) Proposed mechanism of water permeability of hydrophobic membrane i), Janus membrane from hydrophobic Layer‐Et to hydrophilic Layer‐Aq ii), Janus membrane from hydrophilic Layer‐Aq to hydrophobic Layer‐Et iii).

Humidity (or vapor)‐sensitive membranes are a novel class of smart soft materials exhibiting rapid response to vapor concentration or humidity, which have attracted much attentions in terms of multiple functions of humidity‐driven actuating, humidity energy harvesting, biomimetic shape‐memory, etc.^[^ ^2^, ^6^ ^]^ Given the asymmetric wettability and flexible nature of the self‐standing Mem_Janus_, it is of great potential in the application of unidirectional vapor response. The vapor response of the hydrophobic‐hydrophilic membrane at different humidities is shown in **Figure** **4**A–F and Movie S1 (Supporting Information). As shown in Figure 4A–E, when the water vapor rises from bottom to contact the hydrophilic Layer‐Aq face, the membrane curls up obviously at a low humidity of ca. 18% and curls totally when the humidity is increased to ca. 71%. As a contrast, when the hydrophobic Layer‐Et side faces the vapor, the membrane stands still (Figure 4F). Moreover, both faces of the double‐hydrophobic membrane have no response to water vapor (Movie S2, Supporting Information). The phenomena above proved that only the hydrophilic layer has a sensitive response to water vapor and the curling degree is closely related to the humidity (Figure 4G). This specific unidirectional vapor response performance enables the Janus membrane serving as humidity‐driven actuator, soft robot, and breathable skin.

The unidirectional liquid transport of membranes is an essential process in life science and energy‐harvesting field. Driven by the asymmetric differentiation of the wettability between the opposing sides, Janus membranes can improve the transport efficiency and reduce energy consumption in the unidirectional liquid transport process.^[^ ^1^, ^5^ ^]^ Water flux performances of the scale‐up Mem_Janus_ have been characterized under both the gravity‐driven or vacuum pressure‐driven conditions (Figure 4H,I; Figure S26, Supporting Information). Given the pH/temperature‐controllable wettability of the Mem_Janus_, the corresponding variation of the unidirectional water transport performances has been verified. It is noted that for the two double‐hydrophobic membranes prepared at 60 °C (pH = 5) and at pH = 3 (40 °C), both faces are failed to allow water transport. For the hydrophilic‐hydrophobic membranes, water can unidirectionally transport from hydrophobic side (Layer‐Et) to hydrophilic side (Layer‐Aq). For the double‐hydrophilic membranes with asymmetric hydrophilicity, both sides allow water passing through with asymmetric flux. The unidirectional water flux of the Janus membrane prepared at pH = 9 and 40 °C reaches 14 540±290 L (m^−2^ h^−1^ bar^−1^) under negative pressure (0.4 bar). The directional water transport performance of Mem_Janus_ was further confirmed by moisture management test (MMT) (Figure S27, Supporting Information). It was observed from the MMT profiles against time that water dropped on the hydrophobic side (Layer‐Et) rapidly broken through the Janus membrane within 7 s and completely transported to the hydrophilic side (Layer‐Aq) within 92 s. Meantime, water dropped on the Layer‐Aq side was failed to permeate to the Layer‐Et side, further demonstrating the outstanding directional water transport performance of Mem_Janus_.

Furthermore, we have detected the structure and Young's modulus of a typical used membrane (prepared at pH = 9) after negative‐pressure driven water flux test. It can be seen that the used membrane keeps integrate (Figure S28A, Supporting Information), and the morphology keeps well with typical asymmetrical structure (Figure S28B, Supporting Information). The Young's modulus of the used membrane is ≈1.9 GPa (Figure S28C, Supporting Information), revealing its good stability during application for water transport.

It can be seen that the unidirectional water permeability of the Mem_Janus_ is positively related to the asymmetric wettability of the two sides. Due to the asymmetric structure of the Mem_Janus_, noteworthily, the special interpenetrating interface structures also play important roles in unidirectional penetration.^[^ ^35^ ^]^ If water is dropped onto the hydrophobic layer, it takes the form of a meniscus and experiences two opposite forces, namely hydraulic pressure (P_H_) and upward Laplace pressure (P_L_).^[^ ^35^, ^36^ ^]^ The water permeation will be blocked if there is nothing underneath the hydrophobic layer, due to the relatively greater P_L_ formed by the bending water surface (Figure 4J(i)). However, when a hydrophilic layer is underneath the hydrophobic layer, P_L_ will be effectively eliminated due to the water affinity and capillary effect (Pc) of the hydrophilic layer, thus pulling water across the membrane (Figure 4J(ii)). In contrast, the water surface becomes horizontal on the hydrophilic layer, and the corresponding P_L_ is nearly reduced to zero, thus allowing water permeability through the hydrophilic layer under the effect of Pc. However, water permeability will be hindered at the interface of hydrophilic and hydrophobic layers due to the presence of P_L_ on the hydrophobic layer (Figure 4J(iii)).

In consideration of the redox responsiveness of disulfide bond,^[^ ^37^ ^]^ we have further tried to expand the pore channels of the hydrophilic layer via breaking disulfide bonds with the treatment of reductants, thus providing a high potential for enhancing the water flux efficiency. Benefiting from the impermeability from hydrophilic Layer‐Aq to hydrophobic Layer‐Et, the Layer‐Aq of the Mem_Janus_ was soaked in 1 M aqueous solution of dithiothreitol (DTT) for 2 h (see Figure S29A, Supporting Information). As shown in the FT‐IR spectrum of the DTT soaked Mem_Janus_, the characteristic peak for the sulfydryl groups at 2850 cm^−1^ is detected (Figure S29B, Supporting Information), verifying the reduction of disulfide bonds.^[^ ^37^ ^]^ The change in the through‐hole size distribution of the Janus membrane after DTT soaking was measured. As shown in Figure S29D (Supporting Information), the through‐hole size distribution of Mem_Janus_ is relatively uniform in the range of 0.04–0.2 um. After DTT soaking, the pores in the range of 0.04–0.2 um are highly increased, indicating the enlargement of small pores caused by the breakage of disulfide bonds with the treatment of reductants. The pore expanding after DTT soaking is beneficial to improving the water flux efficiency of Mem_Janus_. Moreover, the hydrophilicity of Layer‐Aq is increased due to the appearance of sulfydryl groups, while the hydrophobicity of Layer‐Et keeps well (Figure S29C, Supporting Information). The enhanced wettability differentiation on opposite sides is also favorable to improving the transport efficiency of Janus membranes.^[^ ^2^, ^6^, ^7^, ^8^ ^]^ As expected, the water flux efficiency of the membrane treated by soaking in DTT solution at 40 °C can be highly improved to be 1158±25 L (m^−2^ h^−1^) under natural gravity‐driven condition and 31500±670 L (m^−2^ h^−1^ bar^−1^) under negative pressure‐driven condition (Figure 4I). It is noted that the water flux of the Mem_Janus_ developed in this work is among the highest permeabilities under similar vacuum pressure‐driven conditions (Table S3, Supporting Information).^[^ ^38^, ^39^, ^40^, ^41^, ^42^, ^43^, ^44^, ^45^, ^46^, ^47^ ^]^

## 3 Conclusion

One‐step fabrication of Janus membranes has been achieved via H‐bonded assembly at the oil/water interface, and the precise regulation strategy of the asymmetric surface pattern and wettability has also been demonstrated. PBD‐PBD H‐bonding in oil and water‐PBD H‐bonding in water drive the fabrication of hydrophobic nanospheres and hydrophilic nanofibers, respectively. Water gradient in the interfacial region is crucial for the gradual evolution from nanospheric layer to nanofibric layer, and interface thickness ≥ 2.3 nm is beneficial for the self‐standing nature of Janus membrane. Upon increasing pH in water from pH 3 to 11, an evolution from double‐hydrophobic membrane to hydrophilic‐hydrophobic membrane and then to double‐hydrophilic membrane occurs. Water‐PBD H‐bonding is weakened through increasing temperature or employing urea as a H‐bond competitor or extending folded configuration of PBD, inducing the construction of double‐hydrophobic membranes. The hydrophilic layer of Janus membranes exhibits humidity‐sensitive curling to water vapor. Unidirectional water transportation from hydrophobic to hydrophilic side is achieved, with promising flux of ca. 1158±25 L m^−2^ h^−1^ under gravity and 31500±670 L (m^−2^ h^−1^ bar^−1^) under negative pressure. Our findings highlight an innovative design strategy and regulation mechanism of Janus membrane, providing a versatile pathway for controllable fabrication of multifunctional membrane materials.

## 4 Experimental Section

### Synthesis of 3,3′ – dithiobis(propionyl hydrazine)

Dimethyl 3,3′ – dithiodipropionate (12.01 g, 50.4 mmol) was added into anhydrous methanol (90 mL), followed by the addition of hydrazine hydrate (20.59 g, 403.2 mmol). After stirring at room temperature for 24 h, a white solid (3,3′ – dithiobis(propionyl hydrazine), DTPH) was obtained by centrifugation, and then washed with methanol (30 mL × 2) and diethyl ether (30 mL × 2) and dried in vacuum at 50 °C for 12 h. ^1^H NMR (δ_ppm_, 400 MHz, DMSO‐d6, see Figure S30A, Supporting Information): 9.09 (s, 1H), 4.20 (m, 2H), 2.88 (m, 2H), 2.40 (m, 2H).

### Synthesis of Adipic Dihydrazide

Dimethyl dimethyl adipate (1.74 g, 10 mmol) was added into anhydrous methanol (30 mL), followed by the addition of hydrazine hydrate (4.0 g, 79.9 mmol). After stirring at room temperature for 24 h, a white solid (adipic dihydrazide, ADH) was obtained by centrifugation, and then washed with methanol (30 mL × 2) and diethyl ether (30 mL × 2) and dried in vacuum at 50 °C for 12 h. ^1^H NMR (δ_ppm_, 400 MHz, DMSO‐d_6_, see Figure S30B, Supporting Information): 8.93 (s, 1H), 4.14 (m, 2H), 1.99 (m, 2H), 1.44 (m, 2H).

### Interfacial Assembly of BTA and DTPH at the Water/Oil Interface

1,3,5‐Benzenetricarboxaldehyde (8.1 mg, 0.05 mmol, BTA) was dissolved in 1 mL of ethyl acetate (or toluene, hexane) at 40 °C, and the solution was slowly added into 1 mL of DTPH aqueous solution (18.0 mg, 0.075 mmol) at 40 °C. The biphase system was sealed and standing at 40 °C for a certain time (*i. e*., 1, 3, 6, 12, 24 h). The membrane was taken out slowly and washed with water (1 mL × 3) and ethyl acetate (1 mL × 3), and then dried in vacuum at 70 °C for 12 h. The initial pH of the aqueous phase was 8.1, and was adjusted to be 3, 5, 9, and 11 by using concentrated hydrochloric acid (1 mol L^−1^) and NaOH solution (1 mol L^−1^).

### Assembly of BTA and DTPH in Aqueous Solution

BTA (8.1 mg, 0.05 mmol) was added into 1 mL of DTPH aqueous solution at 40 °C, and the mixture was sealed and standing at 40 °C for 24 h. The membrane was taken out slowly and washed with water (1 mL × 3). The final membrane was then dried in vacuum at 70 °C for 12 h.

### Assembly of BTA and DTPH in Ethyl Acetate

BTA (8.1 mg, 0.05 mmol) was dissolved in 1 mL of ethyl acetate at 40 °C, and DTPH (18.0 mg, 0.075 mmol) was added in the solution at 40 °C. The mixture was sealed and standing at 40 °C for 24 h. The membrane was taken out slowly and washed with water (1 mL × 3). The final membrane was then dried in vacuum at 70 °C for 12 h.

### Assembly of BTA and DTPH in Ethyl Acetate/Water Mixed Solutions

Mixed solutions of ethyl acetate and water with water volume of 2 and 90 vol.% were prepared. Then, BTA (0.0081 g, 0.05 mmol) was dissolved in 1 mL of the mixed solution at 40 °C, and DTPH (18.0 g, 0.075 mmol) was added at 40 °C. The mixture was sealed and standing at 40 °C for 24 h. The membrane was taken out slowly and washed with water (1 mL × 3). The final membrane was then dried in vacuum at 70 °C for 12 h.

### Interfacial Assembly of BTA, Urea and DTPH at the Water/Oil Interface

DTPH (18.0 mg, 0.075 mmol) and urea (450 mg, 0.75 mmol) were mixed in 1 mL of water at 40 °C, and BTA (8.1 mg, 0.05 mmol, BTA) was dissolved in 1 mL of ethyl acetate at 40 °C. The two solutions were mixed slowly, sealed, and standing at 40 °C for a certain time (*i. e*., 1, 3, 6, 12, 24 h). The membrane was taken out slowly and washed with water (1 mL × 3) and ethyl acetate (1 mL × 3), and then dried in vacuum at 70 °C for 12 h.

### Interfacial Assembly of BTA and ADH at the Water/Oil Interface

BTA (8.1 mg, 0.05 mmol) was added into 1 mL of DTPH aqueous solution at 40 °C, and the solution was slowly added into 1 mL of ADH aqueous solution (13.0 mg, 0.075 mmol) at 40 °C. The biphase system was sealed and standing at 40 °C for a certain time (*i. e*., 1, 3, 6, 12, 24 h). The membrane was taken out slowly and washed with water (1 mL × 3) and ethyl acetate (1 mL × 3), and then dried in vacuum at 70 °C for 12 h.

### Characterizations

XRD patterns were acquired in the range of 2θ = 5°−90° with scanning rate of 2°/min on PANayltical Empyrean (Netherlands). The microscopic structures of membranes and powders were intuitively observed by SEM on ZEISS MERLIN CoMPact (Germany). The components in membranes and powders were detected by solid ^13^C NMR spectroscopy (NMR, Bruker Advance 400, Germany). (XPS of the membrane with a thickness of ca. 280 nm etched for different depth down to 150 nm was performed on Axis Supra by using 120 W monochromatic Al Kα radiation (hν = 1486.6 eV), and C 1s peak of contaminant carbon at 284.6 eV was adopted for calibrating the binding energies. FT‐IR (Bruker Vertex 70, Germany) spectra of membranes or powders were recorded on a spectrometer over the wavenumber of 4000 to 400 cm^−1^. AFM was characterized by NanoWizard ®4 using a non‐contact mode cantilever (JPK, Germany). Contact angles with water of the membranes were detected on DSA100 (KRUSS, Germany). The bonding strength between the two sides of the Janus membranes (Layer‐Et versus Layer‐Aq) was tested by conducting a lap shear test. Before test, two Janus membranes with sizes of 40 mm in length and 15 mm in width were prepared and dried under vacuum overnight. Then, the Layer‐Et of one Janus membrane and the Layer‐Aq of another membrane were stacked together with a contact area of 20 mm*15 mm in a moisture atmosphere with a humidity of 50% for 12 h. In the Lap shear test, the combined membranes were stretched in opposite directions with a fixed force of 50 N, and the force‐displacement curve was recorded. The adhesion strength of the two membranes was calculated by the ratio between the tensile force and the contact area of the two membranes. The tensile stress–strain curve of the Janus membrane was measured on an electronic universal testing instrument. The size of the membrane was ≈60 mm in length and 15 mm in width. The clamping distance was 50 mm and the tensile rate was kept at 500 mm min^−1^. The average Young's modulus was reported in parallel test for 5 times. The through‐hole pore size and distribution of Janus membrane before and after DTT soaking were analyzed based on the bubble point method by using a Capillary Flow Porometer (CFP‐1500AE).

### Simulations of Dynamic Covalent Polymer PBD at Water/Oil Interface

Force field parameters for the organic solvents, i.e., EtOAc, hexane and toluene were obtained by the following procedures: the geometry was optimized by using Gaussian 16 at the B3LYP /3‐31G + (d,p) level and then Automated Topology Builder (ATB) to generate the topologies that were compatible with GROMOS force field;^[^ ^48^, ^49^ ^]^ the partial charges were calculated at B3LYP /6‐31G + (d,p) level with CHELPG (CHarges from ELectrostatic Potentials using a Grid based method).^[^ ^50^ ^]^ The geometry for PBD unit was optimized by the methods as shown in next section and the topologies was generated by ATB. The partial charges in PBD were obtained from the RESP charges calculated from Multiwfn.^[^ ^51^ ^]^ SPC model was used for water molecules. All MD simulations were performed using GROMACS package 5.0.7 version with GROMOS force field parameter set 54A7.^[^ ^52^ ^]^ Detailed MD simulations for the assembly performances of PBD in different systems were described in Supplementary Materials.

### Calculation of H‐Bonding

The solvated structures of 3,3′‐dithiobis(propionylhydrazide), adipic dihydrazide and their complexes with water molecules were optimized by using DFT at the level of M06‐2X /def2‐SVP combined with the SMD model method of self‐consistent reaction field (SCRF) theory,^[^ ^53^ ^]^ and the single‐point energies were further calculated at the level of M06‐2X/def2‐TZVP. The charge density topology analysis was performed for the key points (i.e., (3,−1) key points) of interacting bonds by using the atoms‐in‐molecules (AIM) theory. The above calculations were performed on the Gaussian 16 package,^[^ ^48^ ^]^ and the AIM analysis was performed on the Multiwfn program.^[^ ^51^ ^]^ The H‐bond binding energy (BE) was evaluated based on electron density at bond critical point (BCP) using Equation (1):^[^ ^54^ ^]^

(1) $$\mathrm{BE}=-223.08\times \left(\rho_{\mathrm{BCP}}\right)+0.7423$$

where the *ρ* was the electron density of (3,−1) key points.

The Hartree‐Fock (HF) levels with 3–21G basis set were used for modeling structures of the two smallest building blocks in the dynamic covalent polymer PBD, and the structural change upon addition of water molecules.

### Water Vapor Response Performance

Janus membranes with a dimeter of ≈2 cm were formed by the interfacial assembly of BTA and ADH at 40 °C for 24 h. Then, the membrane was fixed with a tweezer and the hydrophobic‐hydrophilic faces were orientated separately to the water vapor at different humidities and room temperature to observe the curly phenomenon.

### Water Permeation Performance

To detect the gravity‐driven water flux performances of the membranes, a membrane device with an effective diameter (D) of 5 cm was employed (Figure S21A, Supporting Information). The water flux (J, L m^−2^ h^−1^) during the gravity‐driven filtration process was calculated according to Equation (2)^[^ ^55^ ^]^:

(2) $$J\;=\frac{▵V}{\mathrm{St}}\;$$

where t was the permeation time (h). The effective area of the membrane (S) was calculated according to the equation of S = π ×  (D/2)^2^ =  1.96 × 10^−3^m^2^. The water volume (ΔV) was 0.02 L with a column height of ≈1 cm during the test.

The water flux under negative pressure condition was tested by using the similar device equipped with a vacuum pump at the downstream of the membrane (Figure S21B, Supporting Information). The penetration time (t) was detected under the pressure of 0.4 bar. The water flux (J, L m^−2^ h^−1^ bar^−1^) was calculated according to Equation (3)^[^ ^43^ ^]^:

(3) $$J\;=\frac{▵V}{\mathrm{St}\Delta P}\;$$

where ΔV was the permeated water volume (0.1 L), S (m^2^) was the effective membrane area (1.96 × 10^−3^m^2^), t (h) was the average penetration time and ΔP(bar) was the operation pressure (0.4 bar). The water flux for each membrane was detected for three times.

Liquid moisture management capability was examined by a Moisture Management Tester (Sdlatlas M290) equipped with bottom and upper sensors. The Janus membrane with the area of 8 cm × 8 cm was dried at 70 °C under vacuum for 24 h prior to the test, and the water transport properties from layer‐Et to layer‐Aq and from layer‐Aq to layer‐Et were tested separately. In the test, water was dropped on the top surface of the membrane and the water content on the top surface and bottom surface were recorded for 120 s by using the upper and bottom sensors.

## Conflict of Interest

The authors declare no conflict of interest.

## Author Contributions

Investigation was done by Y.Q. and Q.M. Formal analysis was done by H.W. and H.X. Writing—original draft was done by Y.Q. Writing—review and editing was done by W.N., L.S., and H.Z. Funding acquisition was done by L.S. and H.Z. Validation was done by T.W. and T.T.

## Supporting information

Supporting Information

Supplementary Movie S1

Supplementary Movie S2

## Data Availability

The data that support the findings of this study are available from the corresponding author upon reasonable request.
