# Supplementary material for: Water‐Assisted Programmable Assembly of Flexible and Self‐Standing Janus Membranes
Source: Adv Sci (Weinh). 2023 Oct 24;10(35):2305239. doi: 10.1002/advs.202305239 (PMC10724425; doi:10.1002/advs.202305239)
Supplement: Supplementary file 1 — Supporting Information [file ADVS-10-2305239-s003.pdf]

ADVANCED SCIENCE
Open Access

Supporting Information

for *Adv. Sci.*, DOI 10.1002/advs.202305239

Water-Assisted Programmable Assembly of Flexible and Self-Standing Janus Membranes

Qun Yi, Mingyue Qiu, Xiaoyu Sun, Haonan Wu, Yi Huang, Hongxue Xu, Tielin Wang, William Nimmo, Tian Tang, Lijuan Shi and Hongbo Zeng**

Supporting Information

Water-assisted programmable assembly of flexible and self-standing Janus membranes

Qun Yi†, Mingyue Qiu†, Xiaoyu Sun†, Haonan Wu, Yi Huang, Hongxue Xu, Tielin Wang, William Nimmo, Tian Tang, Lijuan Shi, Hongbo Zeng**

Prof. Y. Qun, Dr. M. Y. Qiu, Dr. H. N. Wu, Dr. Y. Huang, H. X. Xu, Prof. T. L. Wang, Dr. L. J. Shi
School of Chemical Engineering and Pharmacy
Hubei Key Lab of Novel Reactor & Green Chemical Technology
Key Laboratory of Green Chemical Engineering Process of Ministry of Education
Wuhan Institute of Technology
No.206 Guanggu Road, East Lake New Technology Development Zone, Wuhan 430072, China.
E-mail: shilijuan@tyut.edu.cn

Dr. X. Y. Sun, Prof. H. B. Zeng
Department of Chemical and Materials Engineering
University of Alberta
9211-116 Street NW, Edmonton, Alberta, T6G 1H9, Canada
E-mail: hongbo.zeng@ualberta.ca (H.Z.)

Prof. W. Nimmo
Energy Engineering Group, Energy 2050
University of Sheffield
Western Bank, Sheffield, Sheffield S3 7RD, United Kingdom

Dr. T. Tang
Department of Mechanical Engineering
University of Alberta
9211-116 Street NW, Edmonton, Alberta, T6G 1H9, Canada

Contents

1. Movie Legend.. **4**

Movie S1 This video shows the curling responses of the two faces of hydrophobic-hydrophilic membrane when exposed to an environment with humidity of 69%.. 4

Movie S2 This video shows that there is no visible curling response of the two faces of double-hydrophobic membrane when exposed to an environment with humidity of 70%.................... 4

2. Supplementary Text 5

2.1.MD Simulations for PBD assembly in bulk solutions and at water/oil interface 5

2.2.MD simulations for DTPH assembly in water without or with the presence of urea 9

Scheme S1. Schematic diagram of the structure of traditional Janus membrane and Mem_{Janus} formed by the "bottom-to-up" interfacial assembly in this work.. **11**

Figure S1. Tensile stress versus strain curve of Mem_{Janus} formed through the assembly of BTA (0.05 M) and DTPH (0.075M) at the EtOAc/water interface at 40 °C for 24 h.............. **12**

Figure S2. Morphology and Young's modulus of large-scale Janus membrane...................... **13**

Figure S3. Time-dependent fabrication of Janus membranes **14**

Figure S4. XPS depth profiling for the Janus membrane. **15**

Figure S5. Fabrication and characterization of Janus membrane from the interfacial self-assembly of BTA and DTPH **16**

Figure S6. Variable temperature FT-IR characterization **17**

Figure S7. Molecular model for representing PBD in molecular simulations **18**

Figure S8. Fabrication and characterization of nanomaterials obtained from the self-assembly of BTA and DAO **19**

Figure S9. MD simulation for PBD configurations **20**

Figure S10. PBD configurations for PBD configurations.. **21**

Figure S11. MD simulation for H-bonds.. **22**

Figure S12. SEM images of the membranes formed at the toluene/water interface (a-c) and *n*-hexane/water interface (d) at 40 °C.. **23**

Figure S13. Time evolution for number of H-bonds formed between different components in oil/water interfacial systems containing PBD **24**

Figure S14. Density profiles for PBD, water and oil **25**

Figure S15. (A) Schematic diagram of the lap shear test for the bonding strength of two Janus membranes (Layer-Et vs. Layer-Aq). (B) Lap shear strength (kPa) curve with the increase of displacement (mm)... **26**

Figure S16. Time evolution of change in interaction energy (ΔE) between different components for system with PBD at water/EtOAc interface (sys. #5) **27**
Figure S17. MD simulation result of DTPH in bulk water without and with urea **28**
Figure S18. Radial Distributing Function (RDF) curves from MD simulation **29**
Figure S19. DFT calculation of H-bonding.. **30**
Figure S20. DFT calculation of H-bonding.. **31**
Figure S21. SEM characterization of membrane obtained from the assembly of ADH and BTA **32**
Figure S22. DFT calculated intermolecular H-bonds between protonated DTPH and H_2O... **33**
Figure S23. FT-IR spectra of Mem_{Janus} prepared at different pH (i.e., pH = 3, 5, 9, 11) (A) and Mem_{Janus} after DTT soaking and HCl soaking at different pH (i.e., pH =1, 3, 5).................... **34**
Figure S24. Surface morphology and roughness detected via AFM....................................... **35**
Figure S25. Molar content of DTPH on the opposite sides of the Janus membranes prepared at different pH conditions... **36**
Figure S26. Schematic diagram of water transport devices **..** **37**
Figure S27. Moisture management profiles plotted against time from Layer-Et to Layer-Aq (A) and from Layer-Et to Layer-Aq (B). The blue region and black region in the photos symbolize high water level and low water level, respectively... **38**
Figure S28. Characterization of used membrane (prepared at pH = 9) after water flux detection **39**
Figure S29. Structural characterizations of the Janus membrane after DTT soaking............. **40**
Figure S30. Structural characterizations of building blocks **41**
Table S1 Young's modulus of some membrane materials... **42**
Table S2 Molar content of elements and DTPH on the opposite sides of the Janus membranes prepared at different pH conditions.. **43**
Table S3 Water flux performances of typical reported membranes....................................... **44**
Table S4 MD Simulated systems... **45**
Table S5 MD simulated number of H-bonds between different components at different systems **46**
Table S6 MD simulated thickness of interfacial region **47**
Table S7 MD simulated radius of gyration (R_g), principal radii of gyration (R_x, R_y, R_z), and solvent accessible surface area (SASA) for PBD aggregates in bulk solvents **48**
References **49**

1. Movie Legend

Movie S1. This video shows the curling responses of the two faces of hydrophobic-hydrophilic membrane when exposed to an environment with humidity of 69%

Movie S2. This video shows that there is no visible curling response of the two faces of double-hydrophobic membrane when exposed to an environment with humidity of 70%

2. Supplementary Text

2.1.MD Simulations for PBD assembly in bulk solutions and at water/oil interface.

Model details. Force field parameters for ethyl acetate (EtOAc) were obtained by the following procedures: the geometry was optimized by using Gaussian 16 at the B3LYP /6-31G(d,p) level and then Automated Topology Builder (ATB) to generate the topologies that were compatible with GROMOS force field [1,2]; the partial charges were calculated with CHELPG (CHarges from ELectrostatic Potentials using a Grid based method) [3]. Topologies for hexane were generated from ATB and the partial charges were adopted from the previous literatures [4] for an n-alkane (heptane). Topologies and partial charges for toluene were directly adopted from the literatures [4]. A repeating unit (as shown in Fig. S7) was built to represent the supramolecular PBD, where the important features, such as the three-branched structure and functional groups, were kept. The force field parameters for the PBD unit were obtained as shown in the main text. It should be noted that there are limitations to simulate the supramolecular structure PBD at molecular level due to the scale differences. The aim of molecular simulations is to provide analysis of the H-bonding in various environment, adsorption at different interfaces, and assemblies for model PBD units, excluding the morphologies of microstructure assemblies.

Simulation details. Molecular dynamics (MD) simulations were performed on 3 series of systems. Each series had the same construction of simulation box with different solvents. Prior to the construction of simulations box of sys. 1-4 (see Table S4), PBD molecules were randomly dispersed in a cubic box with dimensions of 12 nm ×12 nm × 12 nm, the box centered in a box 16 nm ×16 nm × 16 nm, aiming to obtain the equilibrated PBD assembly in vacuum without interacting with the neighboring periodic boxes. The pre-equilibrated PBD aggregate was centered in a box of 16 nm ×16 nm × 16 nm which was then solvated with EtOAc (sys. 1), toluene (sys. 2), hexane (sys. 3), or water (sys. 4) molecules, respectively. After a production run for 100 ns, the final configurations for sys. 1-4 in replica 1 were obtained. In replica 2 for sys. 1-4, the PBD molecules were randomly dispersed in a box of 12.5 nm × 12.5 nm × 12.5 nm and then solvated by different organic solvents. The final configurations were obtained after a 40 ns of simulation. Both replicas agreed on the aggregation of PBD in different solvents, while the molecules might interact with the molecules in the neighboring periodic boxes and restrained the further aggregation in replica 2. Thus, the results from replica 1 were used for the

data analysis. Two additional system with PBD in bulk water/EtOAc (2 : 98 vol. %) mixture and water/EtOAc (90:10 vol. %) was simulated with the method for replica 1. The results of H-bonds formations for systems with PBD in bulk solvents are shown in Fig. S11.

For systems in the second series (sys. 5-7, see Table S4), a box of 12 nm × 12 nm × 10 nm was filled with water molecules and then centered in a box of 12 nm × 12 nm × 28.2 nm. PBD molecules were arranged as two 4×8 arrays near the surface of water box. The simulation box was then solvated with EtOAc (sys. 5), toluene (sys. 6), hexane (sys. 7), respectively. The density profiles for all components in sys. 5-7 are shown in Fig. S14. The bulk density for EtOAc, toluene, and hexane was obtained at z in 10 nm-15 nm where the density of water and PBD were negligible. The average density for bulk EtOAc, toluene, and hexane were 864 kg/m^3, 853 kg/m^3, and 648 kg/m^3 at 313.15 K, which was in good agreement with the experimental data of 870 kg/m^3 at 318. 15 K [5], 848 kg/m^3 at 313.15 K [6], and 642 kg/m^3 at 313.15 K [5], respectively.

The third series (sys. 8-10, see Table S4) were the control systems with pure organic solvent and water interfaces. Each system underwent an energy minimization, 100 ps NVT equilibration, and 40 ns production simulations with $NP_{normal}AT$ ensemble at temperature of 313.15 K with the same parameters as shown in the main text.

Equilibration of systems. The 3D visualization of final configurations was performed in VMD in perspective view, as shown in Fig. S10 for sys. 1-3 and Fig. S9D for sys. 4. The results for system of PBD in water/EtOAc (2 : 98 vol. %) are shown in Fig. S9A-C. Time evolution for number of H-bonds in pure oil phases (sys. 1-3) and water (sys. 4) are shown in Fig. S11, where the curves reach plateau during 30-40 ns. Density profiles averaged over every 1 ns during the last 10 ns for sys. 5-7 with interfaces are shown in Fig. S14A-C. Ten curves for each component, i.e., PBD, water or organic solvent show good convergence, suggesting that the systems are well equilibrated during the last 10 ns. Each density profile curve contains a solid line as the average values and the shades between the minimum to maximum values over the last 10 ns. Due to the good convergence, the shade along with each curve has been negligible. Time evolution for the number of H-bonds in the oil/water interfacial systems with the presence of PBD molecules (sys. 5-7) are shown in Fig. S13. The average number of H-bonds every 10 ns from 30 ns to 80 ns are shown along with each curve. During the last 10 ns, the systems are well equilibrated as each curve reaches a plateau with random fluctuation. Thus, the last 10 ns of each simulation has been used for the calculation of H-bonds as shown in Table S5. The error

bars of the number of H-bonds have been added for all the systems, and all the values for number of H-bonds are shown in the format of “average (standard deviation)”.

Shape of PBD aggregates in bulk solvents. To gain quantitative analysis on the shape of PBD aggregates in bulk solvents. the radius of gyration (R_g) about the center of mass (COM) and the principal radii of gyration were calculated by Gromacs, as:

$$R_g = \left(\frac{\sum_i m_i {r_i}^2}{\sum_i m_i}\right)^{0.5} \quad (1)$$

where m_i and r_i are, respectively, the mass and distance from the COM for atom i. The principal axes passing through the COM are denoted as x, y, and z. Principal radii of gyration about the axes are calculated as:

$$R_x = \left(\frac{\sum_i m_i ({y_i}^2+{z_i}^2)}{\sum_i m_i}\right)^{0.5} \quad (2)$$

$$R_y = \left(\frac{\sum_i m_i ({x_i}^2+{z_i}^2)}{\sum_i m_i}\right)^{0.5} \quad (3)$$

$$R_z = \left(\frac{\sum_i m_i ({x_i}^2+{y_i}^2)}{\sum_i m_i}\right)^{0.5} \quad (4)$$

where (x_i, y_i, z_i) are the coordinates for atom i and the summations are over all the atoms in the aggregates. The average values over the last 10 ns for R_g, R_x, R_y, R_z are shown in Table S5. The difference between R_x, R_y, R_z indicated the shape of the aggregates that a sphere like structure had $R_x \approx R_y \approx R_z$, 1D structure had $R_x \ll R_y \approx R_z$ and a short cylinder had $R_x < R_y \approx R_z$. While in sys. 1-4, the principal radii of gyration followed $R_x < R_y < R_z$, suggesting a similar shape of assemblies in different bulk solvents due to the scale of molecular model. The overall R_g and the solvent accessible surface area (SASA) slightly decreased as the solvent changed in sys. 1 to sys. 4. It indicated that the aggregates shape became more compact, and the surface of aggregates became less accessible, which were subject to the specific molecular model used in the work. For example, the model molecule had the three branched structure and a small molecule size, resulting a higher ratio of the tails in the whole molecules. The tails tended to interact with EtOAc, which made the surface of PBD assembly less compact. It was expected that the supramolecular PBD had much less ratio of tails and the morphology could be different. Though the microscopic structure supramolecular PBD assemblies could not be modeled, the simulations of sys. 1-4 confirmed that the H-bonds formation of PBD assemblies in various solvents were greatly dependent on the solvent type.

In the additional systems (Fig. S11), some water molecules were dispersed in EtOAc in the system with PBD in bulk water/EtOAc (2 : 98 vol. %) mixture and some EtOAc molecules were dispersed in water in the system with PBD in water/EtOAc (90:10 vol. %) mixture, which was consistent with inter-solubility of water and EtOAc. As water molecules partially accumulated in the system with PBD in water/EtOAc (2:98 vol.), water/EtOAc interface was formed and part of the PBD assembly was located at the interface. With the presence of interface, the shape of PBD assembly was altered that R_g was greater in the system with water/EtOAc (2:98 vol.). Similarly, the PBD assembly had the greater R_g and SASA in the system with PBD in water/EtOAc (90:10 vol. %). Interestingly, the principal radii of gyration of PBD assembly followed $R_x < R_y \approx R_z$ in the systems with water/EtOAc mixture, suggesting a cylinder-like shape. With the increasing water ratio from water/EtOAc (2:98 vol.) to water/EtOAc (90:10 vol.), the different between R_y $(or\ R_z)$ and R_x became more significant. It confirmed that the formation of water/EtOAc interface would alter the shape of PBD assembly.

Calculation of interfacial region. Density profile $\rho(z)$ is a function of z, where the simulation box is sliced along z-direction and the density in each slice is calculated. For each system, there were two interfaces, where the water and organic solvent coexisted and the curves for water and organic solvent intersected at certain z values. The bulk phase was defined as that the mass ratio of water or organic solvent was over 99%. Thus, the interfacial region was defined as that both water and organic solvent have less than 99% mass ratio. The criteria are expressed as follows:

$$\begin{cases} \frac{\rho(z)_{water}}{\rho(z)_{total}} \geq 99\%,\ z\ in\ bulk\ water\ phase; \\ \frac{\rho(z)_{organic}}{\rho(z)_{total}} \geq 99\%,\ z\ in\ bulk\ organic\ phase; \\ \frac{\rho(z)_{organic}}{\rho(z)_{total}} < 99\%\ and\ \frac{\rho(z)_{water}}{\rho(z)_{total}} < 99\%,\ z\ in\ interfacial\ region; \end{cases} \quad (5)$$

where $\rho(z)_{total} = \rho(z)_{water} + \rho(z)_{organic}$ for control systems without PBD and $\rho(z)_{total} = \rho(z)_{water} + \rho(z)_{organic} + \rho(z)_{PBD}$ for systems with PBD. As an example, for one of the interfaces (z around 5 nm) as shown in the top panel of Fig. S10, with z in range 4.1 nm to 7.8 nm, the density profiles met the criteria $\frac{\rho(z)_{EtOAc}}{\rho(z)_{total}} \leq 99\%\ and\ \frac{\rho(z)_{water}}{\rho(z)_{total}} \leq 99\%$. When z was lower than the left boundary (4.1 nm), $\frac{\rho(z)_{water}}{\rho(z)_{total}} > 99\%$, indicating a bulk water phase. And when z>7.78 nm, $\frac{\rho(z)_{EtOAc}}{\rho(z)_{total}} > 99\%$, suggesting a bulk EtOAc phase. Similarly, the boundaries of the other interface (z around 16.0 nm) were found to be 12.2 nm and 16.0 nm. The thickness

for each interface is the difference of the boundaries and the reported values were the average thickness of two interface, as summarized in Table S6.

Gradient region of water. The gradient region of water in z-direction is defined as where the water density is within 1% - 99% of its bulk density. The plateau of water density where z is less than 2 nm and greater than 18 nm is used as the bulk region, and the average density in the bulk region represents the bulk density of water.

Due to the difference in the scale between the realistic and simulated systems, the final configurations from the simulations represent the stable equilibrated state of the ideal systems, which are not aimed to capture the kinetics of the assembly process and the real assembly arrangement. In MD simulations, the relations between PBD assemblies and the contribution of H-bonds between different groups are discussed, aiming to provide mechanistic understanding on the assembly performance in different environments.

Interaction energies in the system with PBD at water/EtOAc interface. The time evolution of change in interaction energies (ΔE) between different components in the system with PBD at water/EtOAc interface are plotted against the simulation time as shown in Figure S16. The short-range Columbic interaction energy and short-range Lennard-Jones energy were considered and calculated by gmx energy in GROMACS.[7–9]

Summary. The MD simulations investigated the assembly of PBD and the H-bond formation of the systems with PBD in bulk solvents and at organic solvent/water interfaces. Firstly, the difference in the shape of PBD assemblies in pure solvents were subject to the feature of the simulated PBD unit model. Secondly, in the mixtures of water/EtOAc, the presence of water/EtOAc interface altered the shape of PBD assemblies, which tended to form cylinder-like structure rather than globule-like in pure solvents. Thirdly, H-bond formation within PBD assembly (PBD-PBD H-bonds) were substituted by the H-bonds formation between PBD and water (compare sys. 4 with sys. 1-3), which fundamentally suggested that the conformation of PBD assembly would be altered when specific groups of atoms (H-bonds acceptors and donors) formed H-bonds with water. Lastly, the thickness of PBD layer at the interface decreased when solvent changed from EtOAc to toluene and heptane, probably due to the solubility of PBD in different organic solvents. The thinner PBD layer contributed to more interaction between PBD and water, indicated by the increasing of H-bond formation.

2.2.MD simulations for DTPH assembly in water without or with the presence of urea.

The force field parameters for DTPH and urea were obtained from the same procedure for organic solvents. The simulation procedures were the same as that for PBD in bulk solutions. A system of 360 DTPH molecules in bulk water (simulation box size: 12 nm × 12 nm × 12 nm) without urea was simulated for 40 ns. Water molecules were then removed from the equilibrated system, 900 urea molecules were inserted randomly, and the simulations box was solvated by water again, which formed the initial configuration of the system with urea.

2.Supplementary Figures:

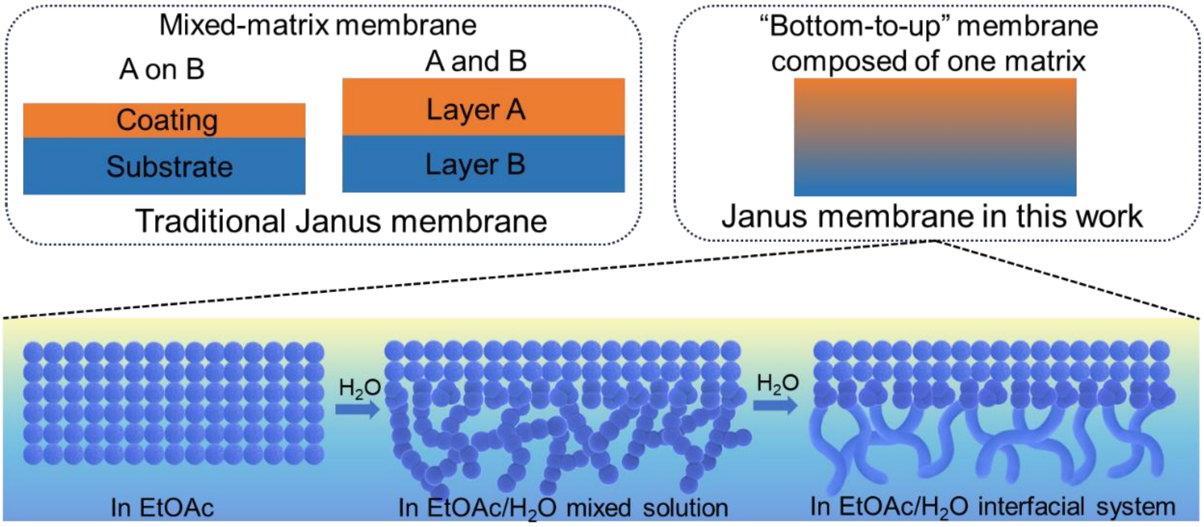

Scheme S1. Schematic diagram of the structure of traditional Janus membrane and Mem_{Janus} formed by the "bottom-to-up" interfacial assembly in this work.

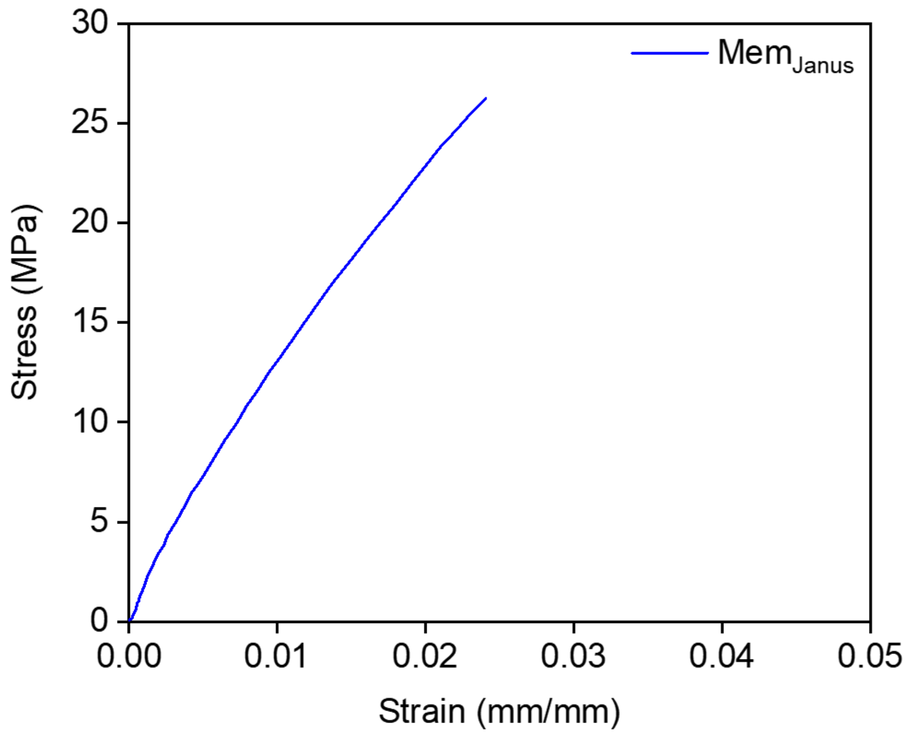

Figure S1. Tensile stress versus strain curve of Mem$_{Janu}$ formed through the assembly of BTA (0.05 M) and DTPH (0.075M) at the EtOAc/water interface at 40 °C for 24 h.

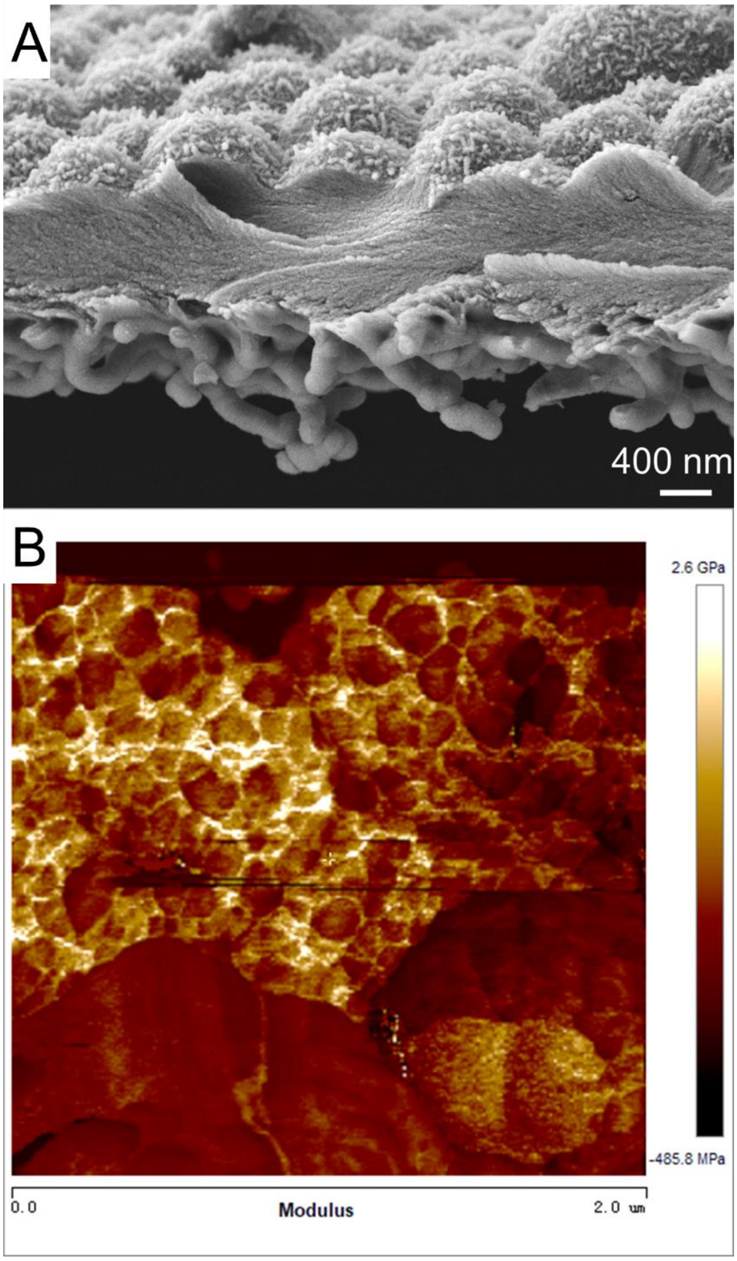

Figure S2. Morphology and Young's modulus of large-scale Janus membrane. (A) SEM image of the cross section of large-scale freshly prepared Mem_{Janus} and (B) Young's modulus of large-scale Mem_{Janus} stored under room condition for eight months. The membrane was formed through the assembly of BTA (0.05 M) and DTPH (0.075M) at the EtOAc/water interface at 40 °C for 24h.

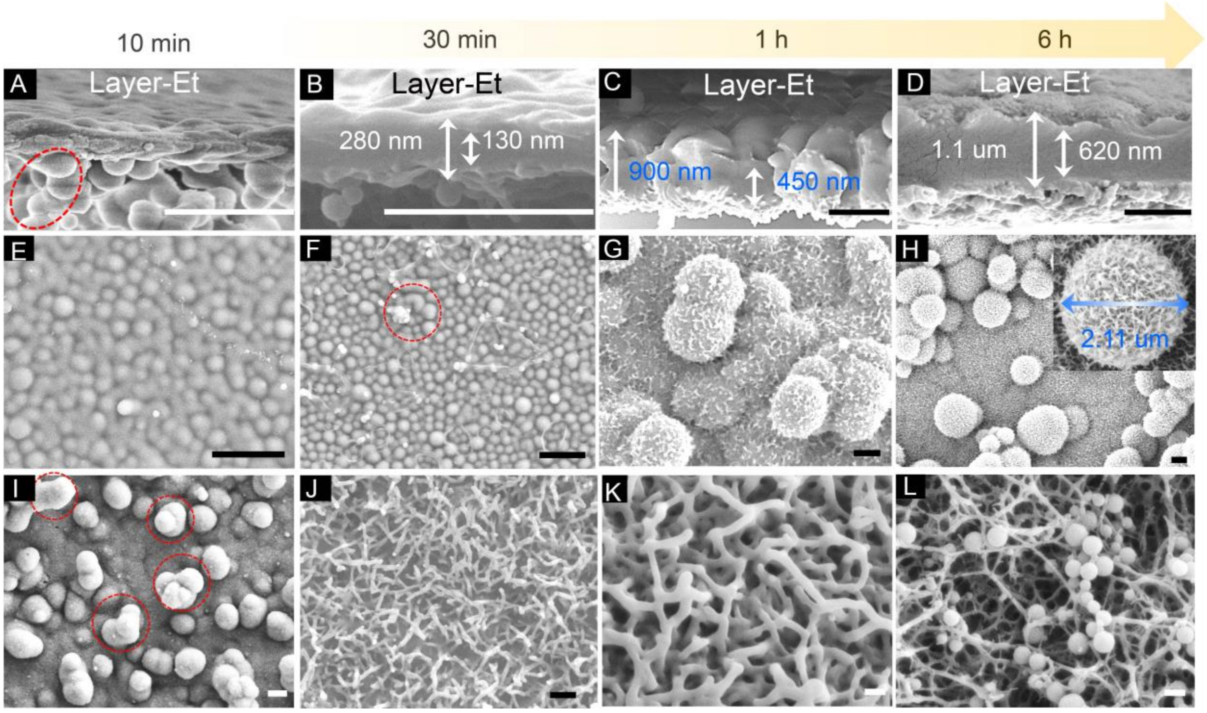

Figure S3. Time-dependent fabrication of Janus membranes. SEM images of the Mem_{Janus} formed at different time: 10 min (A,E,I), 30 min (B,F,J), 1 h (C,G,K) and 6 h (D,H,L). (A-D) Cross section; (E-H) Layer-Et; (I-L) Layer-Aq. Concentrations of BTA and DTPH are 0.05 M and 0.075 M, respectively. Scale bars: (A-H) 1 um, (I-L) 200 nm.

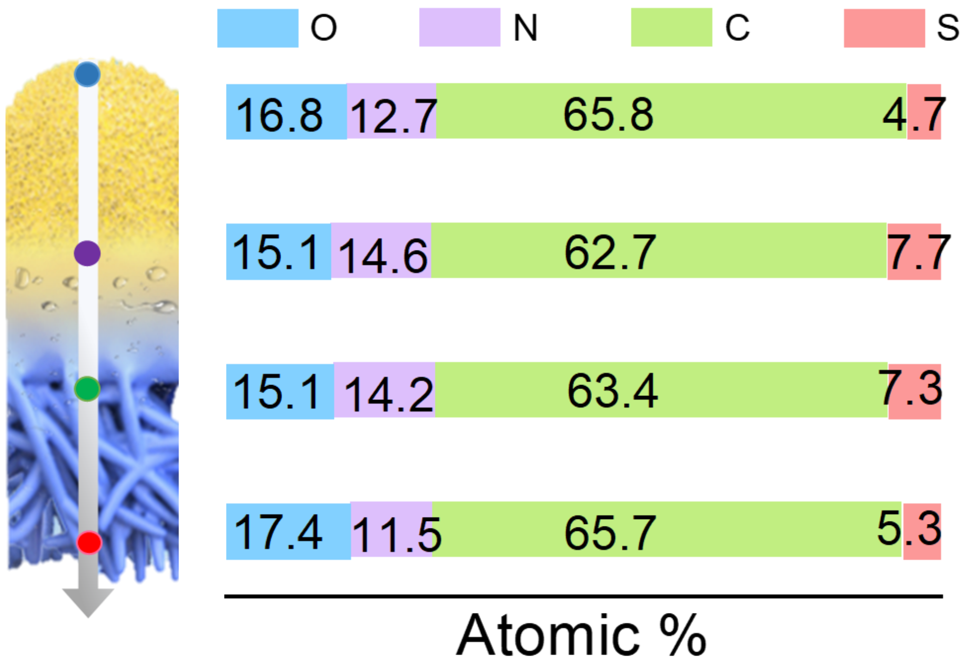

Figure S4. XPS depth profiling for the Janus membrane. The atomic content of O, N, C and S obtained from the XPS depth profiling down to 150 nm for the Mem_{Janus} formed at 30 min.

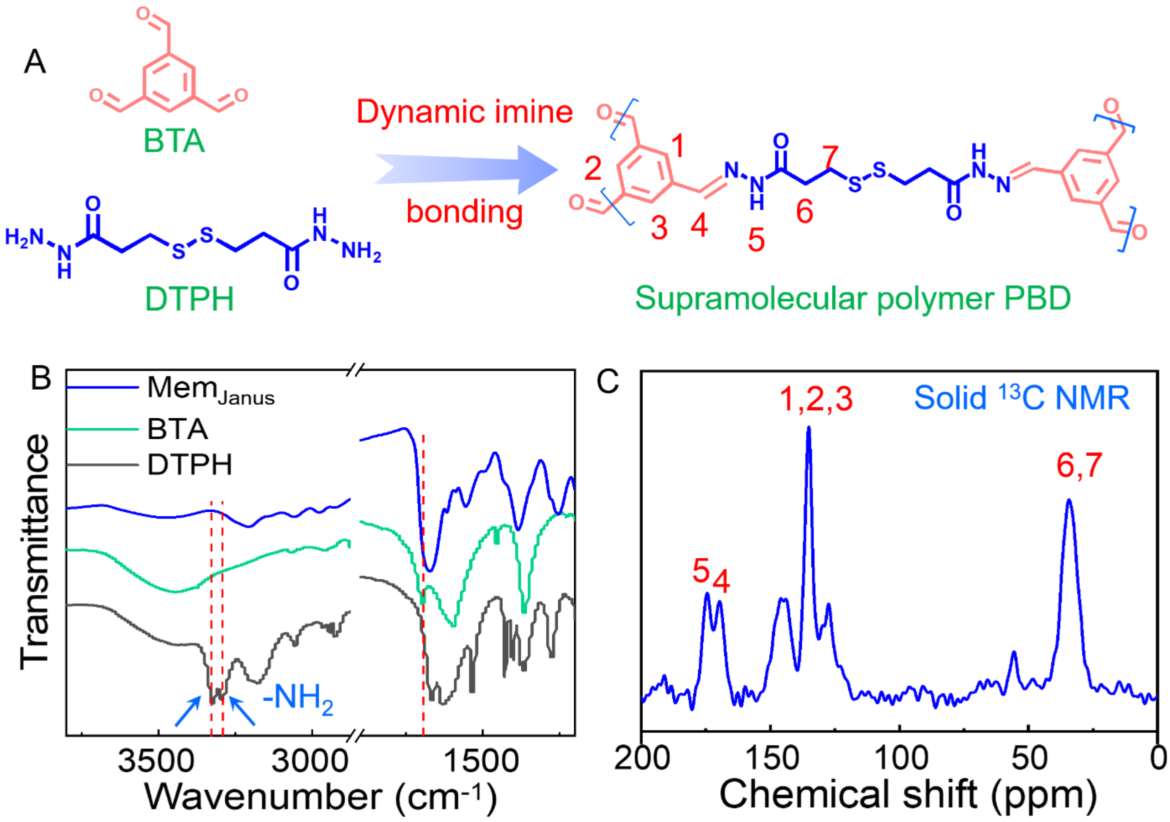

Figure S5. Fabrication and characterization of Janus membrane from the interfacial self-assembly of BTA and DTPH. (A) Schematic diagram of interfacial self-assembly of 1,3,5-benzenetricarboxaldehyde (BTA) and 3,3' - dithiobis(propionyl hydrazine (DTPH) at the ethyl acetate (EtOAc)/water interface at 40 °C. (B) FT-IR spectra of Mem_{Janus}, BTA and DTPH. (C) Solid ^{13}C MAS NMR spectrum of Mem_{Janus}.

The FT-IR spectrum of the Janus membrane proves the absence of the characteristic peaks for the amino group at 3325 cm^{-1} and 3291 cm^{-1}, and meanwhile, the signal of benzoic imine group at 169 ppm appears on the solid ^{13}C NMR profile of the Janus membrane. This phenomenon verifies that the amino group on DTPH and aldehyde group on BTA have been reacted into dynamic benzoic imine group, thus inducing the construction of supramolecular polymer PBD.

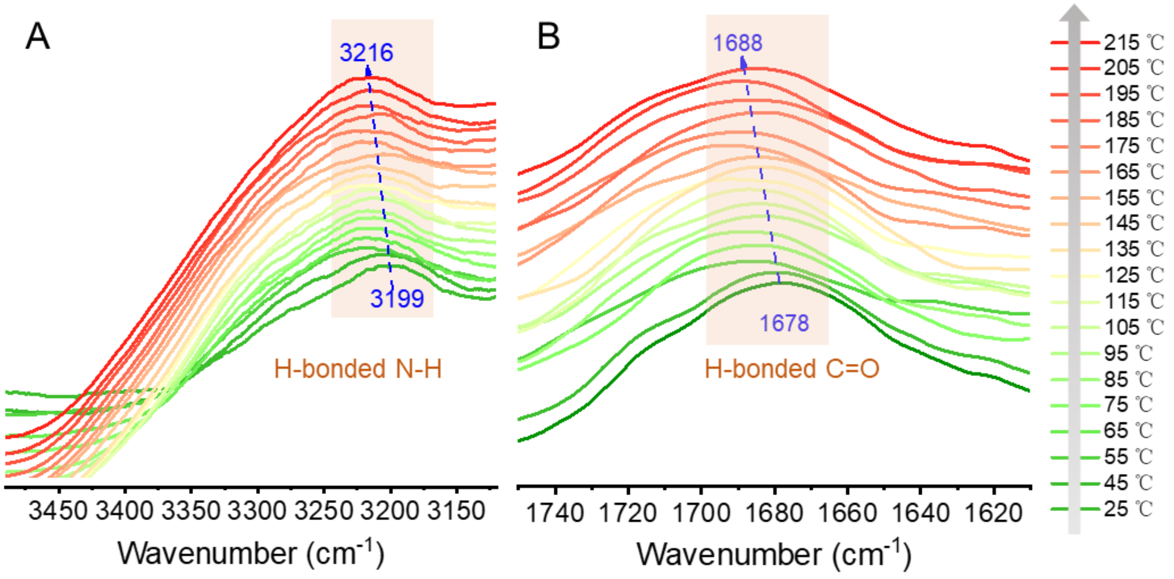

Figure S6. Variable temperature FT-IR characterization. Variable temperature FT-IR spectra of the nanospheres formed in absolute EtOAc solution from 25 °C to 225 °C in the $\nu_{N\text{-}H}$ (A) and $\nu_{C=O}$ (B) regions.

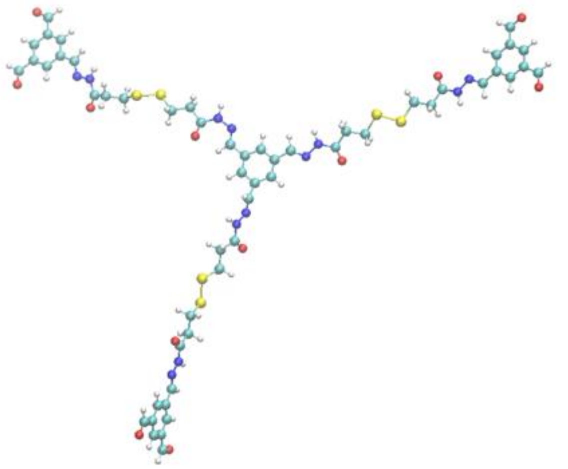

Figure S7. Molecular model for representing PBD in molecular simulations. Shown in ball and stick: cyan - C, blue - N, red- O, yellow - S and white - H.

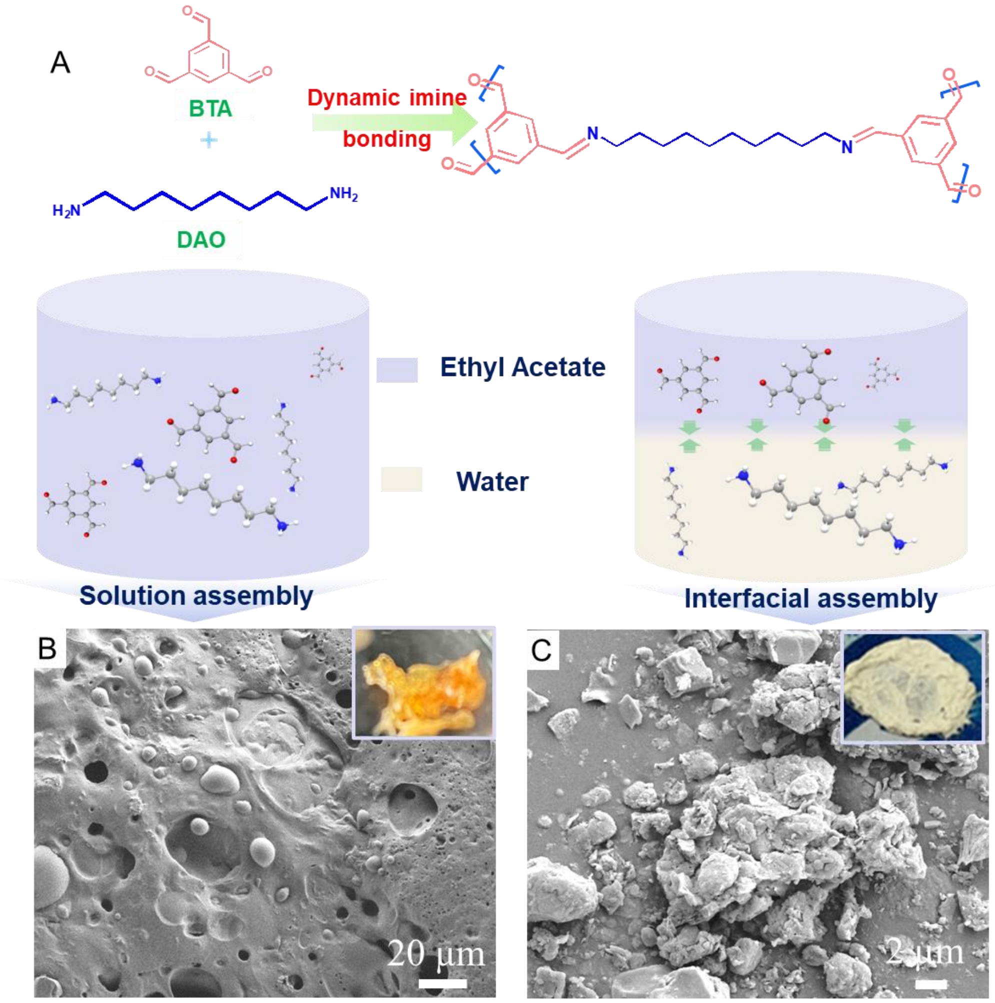

Figure S8. Fabrication and characterization of nanomaterials obtained from the self-assembly of BTA and DAO. (A) Schematic diagram of self-assembly of 1,3,5-benzenetricarboxaldehyde (BTA) and diaminooctane (DAO) in absolute EtOAc solution or at the EtOAc-H_2O interface at 40 °C. (B,C) SEM images of the product formed in absolute EtOAc solution (B) and at the EtOAc-H_2O interface (**C**). Concentrations of BTA and DAO are 0.05 M and 0.075M, respectively.

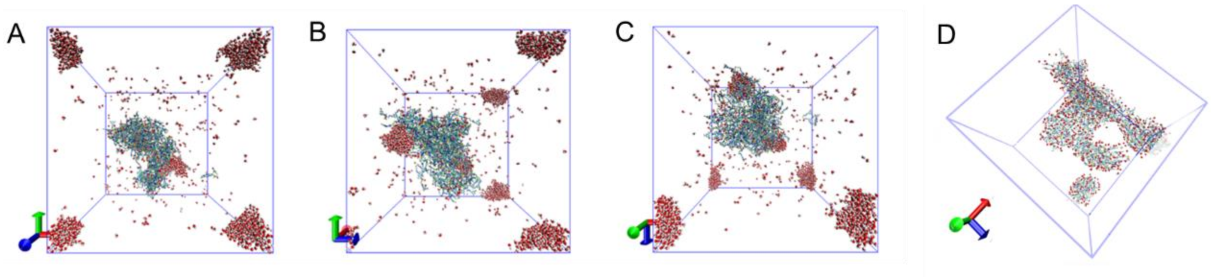

Figure S9. MD simulation for PBD configurations. PBD configurations in EtOAc/water (2 vol.%) mixed system (**A-C**) and bulk water (**D**). From left to right are the views along z axis (blue), x axis (red) and y axis (green). Bonds between carbon (cyan color), oxygen (red color) and nitrogen (blue color) atoms: PBD, organic solvents are removed for clarity.

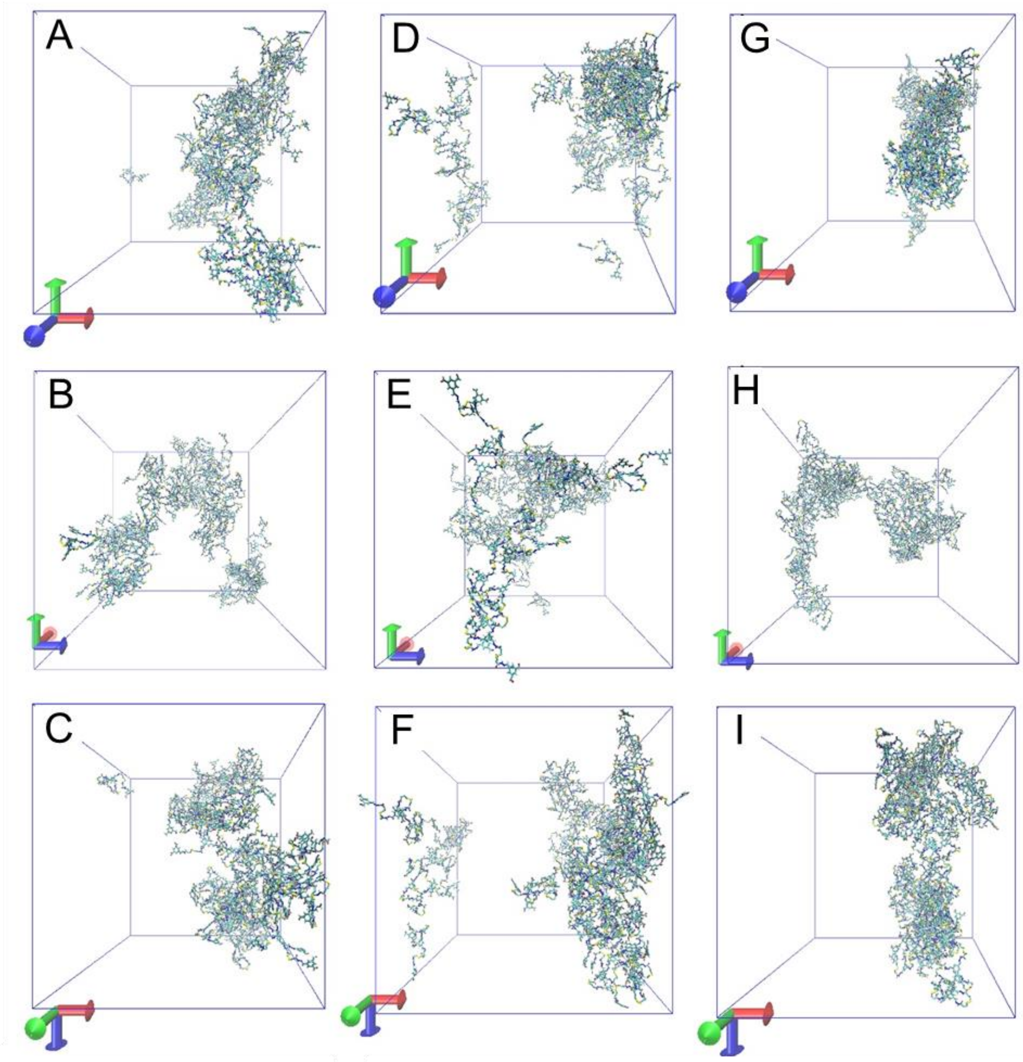

Figure S10. PBD configurations for PBD configurations. PBD configurations in bulk EtOAc (A-C), toluene (D-F) and hexane (G-I). bonds between carbon (cyan color), oxygen (red color) and nitrogen (blue color) atoms: PBD, organic solvents are removed for clarity. From upper to down are the views along z axis (blue), x axis (red) and y axis (green).

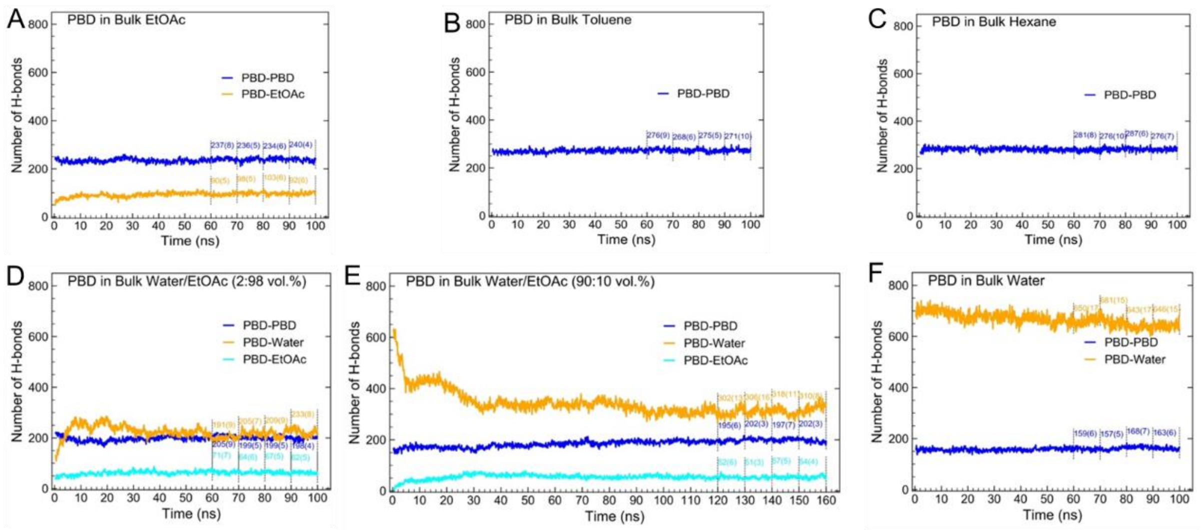

Figure S11. MD simulation for H-bonds. Time evolution for number of H-bonds in EtOAc/water mixed systems and bulk solvents. Systems: EtOAc (A), toluene (B), hexane (C), water (2 vol.%)/EtOAc mixed system (D), water (90 vol.%)/EtOAc mixed system (E), and bulk water (F).

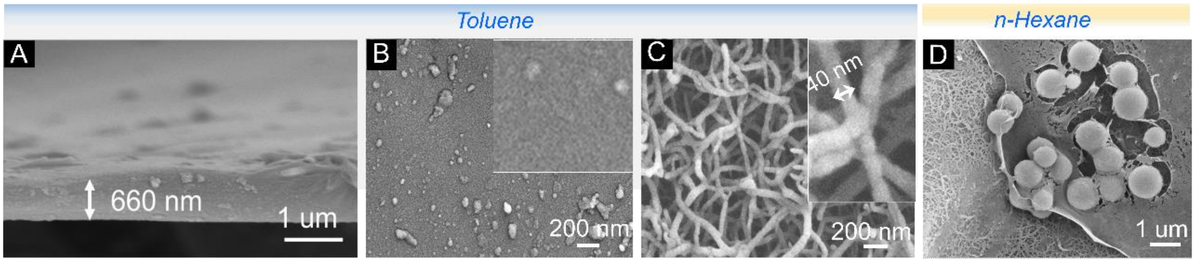

Figure S12. SEM images of the membranes formed at the toluene/water interface (a-c) and *n*-hexane/water interface (d) at 40 °C; (A) Cross section, (B) Layer-Tol, (C) Layer-Aq.

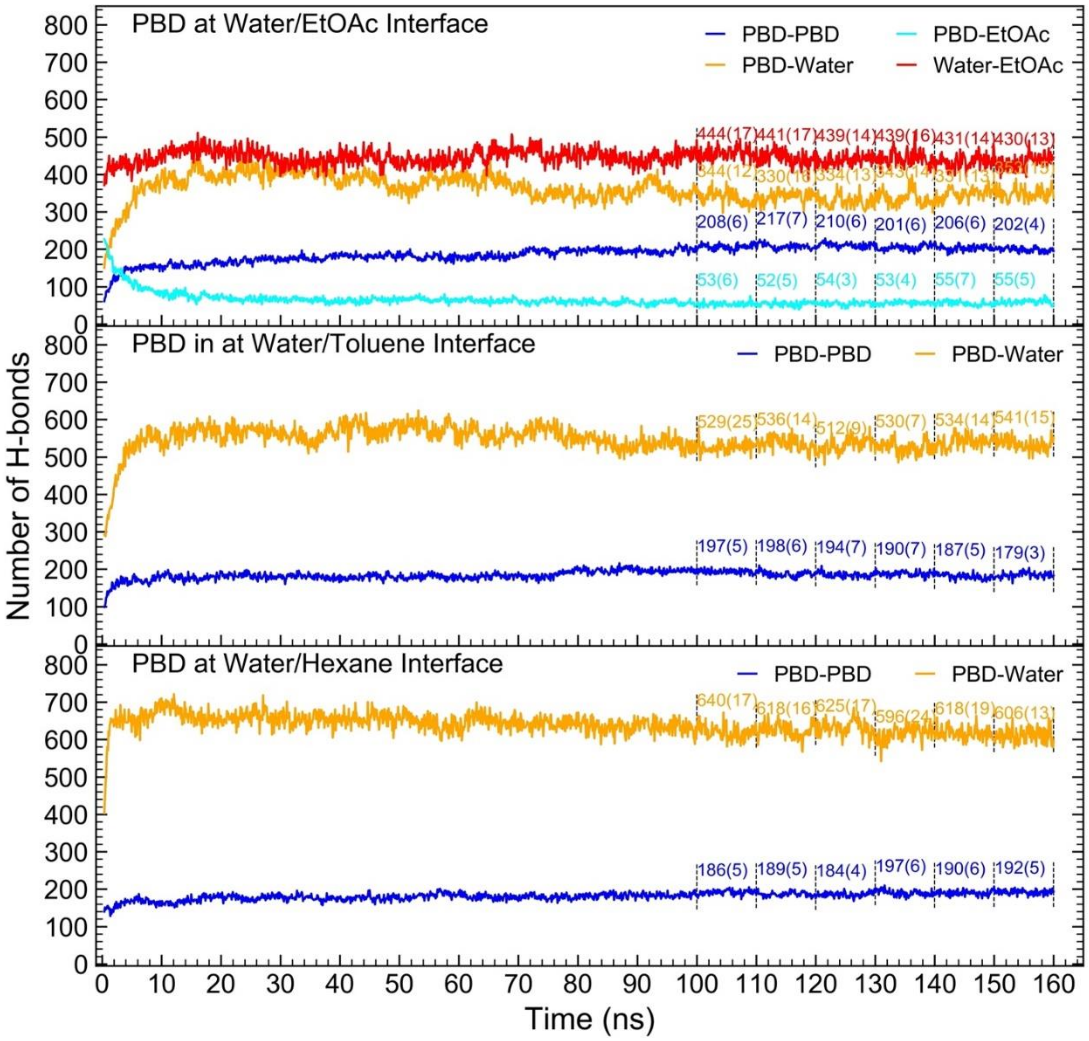

Figure S13. Time evolution for number of H-bonds formed between different components in oil/water interfacial systems containing PBD. Systems: water/EtOAc interface (top), water/toluene interface (middle) and water/hexane interface (bottom). Values are the average with standard deviation (shown in parenthesis) during every 10 ns.

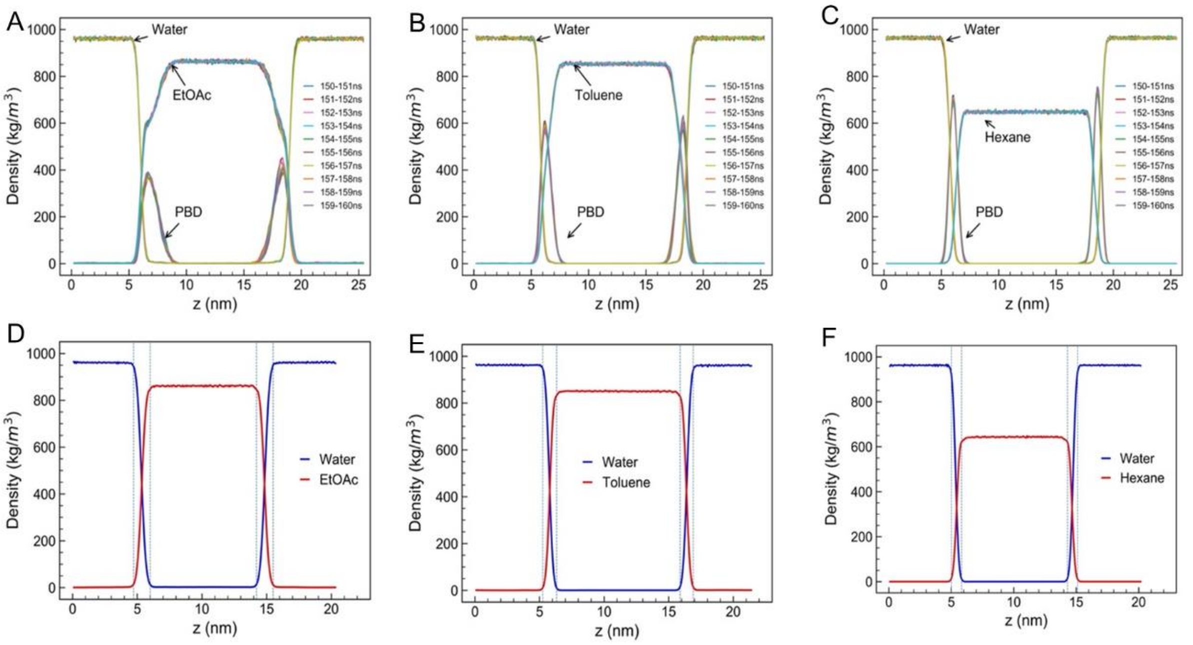

Figure S14. Density profiles for PBD, water and oil. (A-C) Density profiles averaged over every 1 ns during the last 10 ns for PBD, water, and organic solvents in PBD-contained interfacial systems of EtOAc/water (A), toluene/water (B) and hexane/water (C). (D-E) Density profiles of organic solvents (i.e., EtOAc, toluene, hexane) and water averaged over the last 10 ns for control systems of EtOAc/water (D), toluene/water (E) and hexane/water (F).

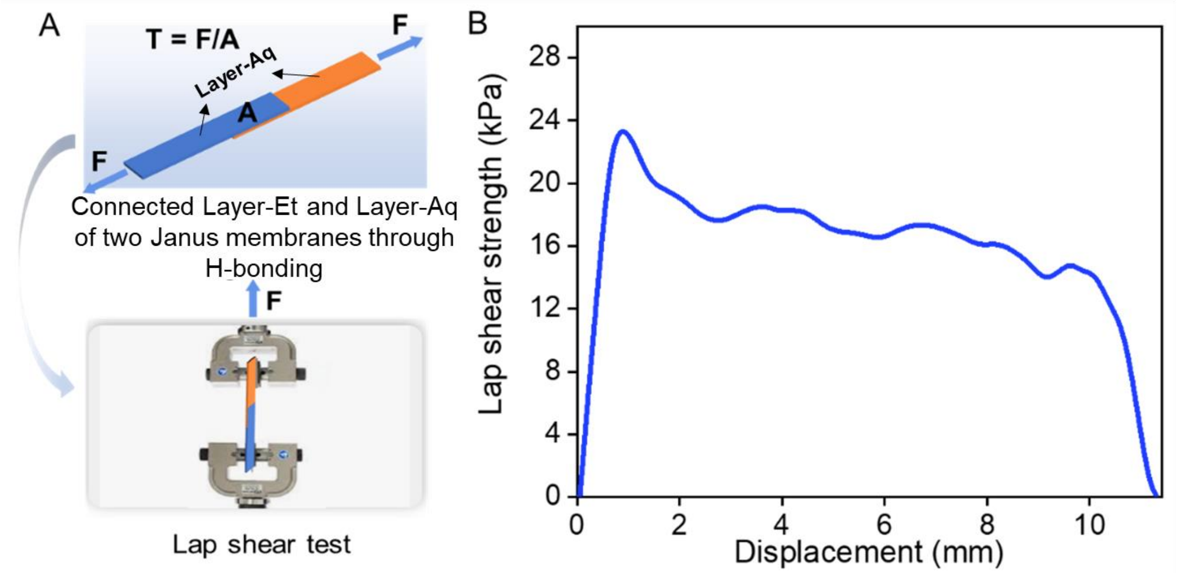

Figure S15. (A) Schematic diagram of the lap shear test for the bonding strength of two Janus membranes (Layer-Et vs. Layer-Aq). (B) Lap shear strength (kPa) curve with the increase of displacement (mm).

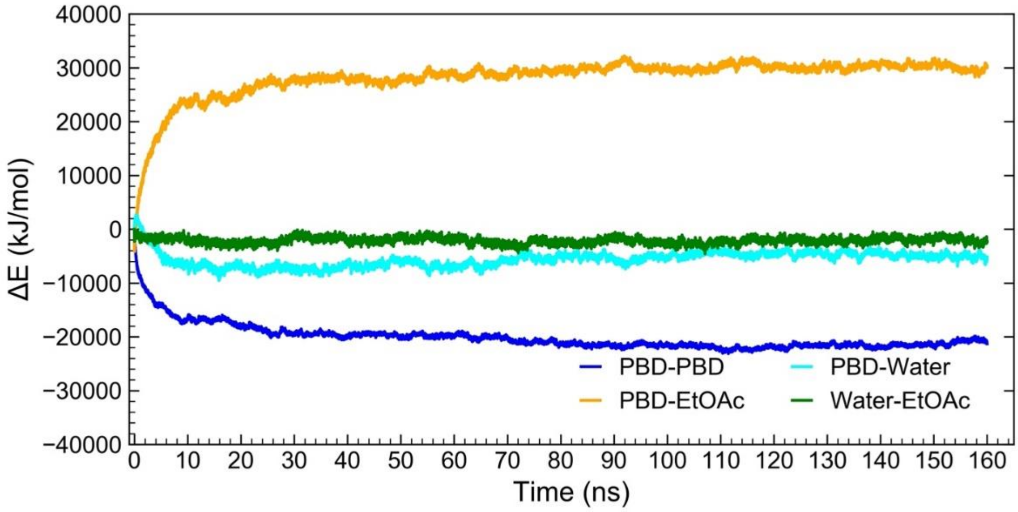

Figure S16. Time evolution of change in interaction energy (ΔE) between different components for system with PBD at water/EtOAc interface (sys. #5).

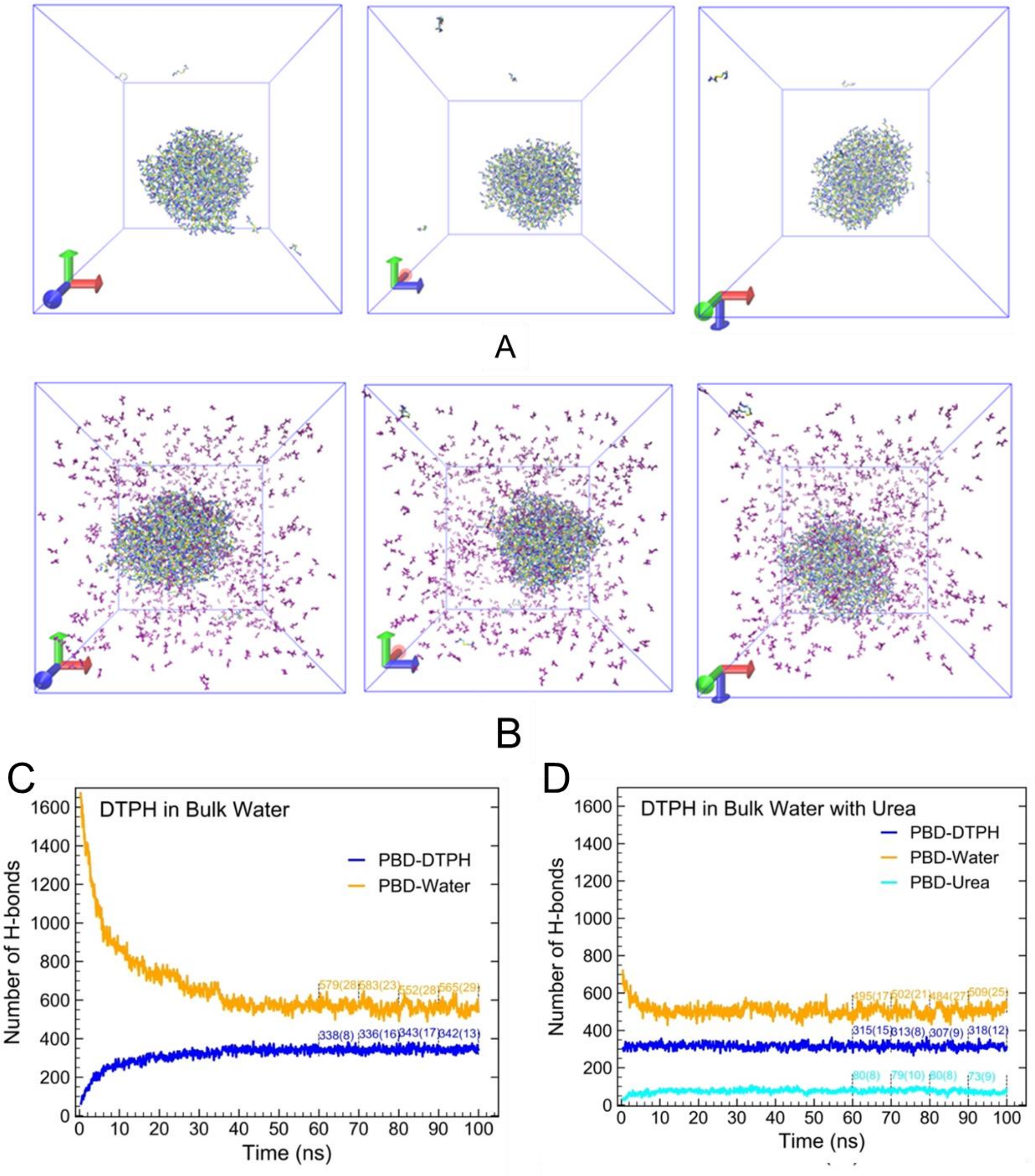

Figure S17. MD simulation result of DTPH in bulk water without and with urea. Final configurations of DTPH in water without urea (A) and with urea (B). (Bonds between carbon (cyan color), oxygen (red color) and nitrogen (blue color) atoms: DTPH; purple bonds: urea; water molecules are removed for clarity. From left to right are the views along z axis (blue), x axis (red) and y axis (green). Time evolution of H-bonds between different components in systems with DTPH in bulk water without urea (C) and with urea (D). (Values along each curve were the average number of H-bonds in the last 10 ns with standard deviation in parenthesis.)

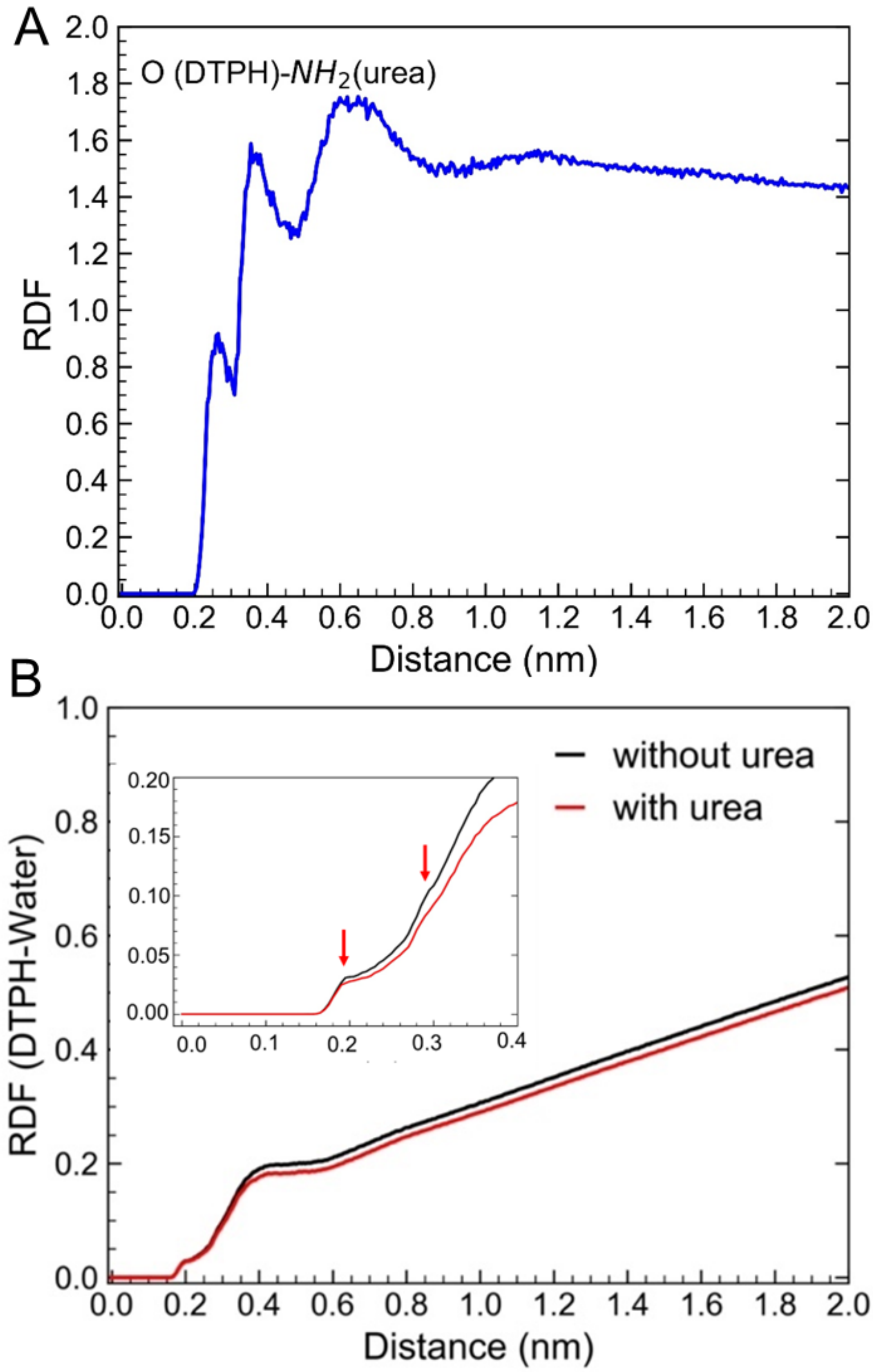

Figure S18. Radial Distributing Function (RDF) curves from MD simulation. (A) RDF curve of amine (NH_2) in urea with respect to oxygen atoms in DTPH. (B) RDF curve of water with respect to all atoms in DTPH in systems without (black) and with (with) the present of urea.

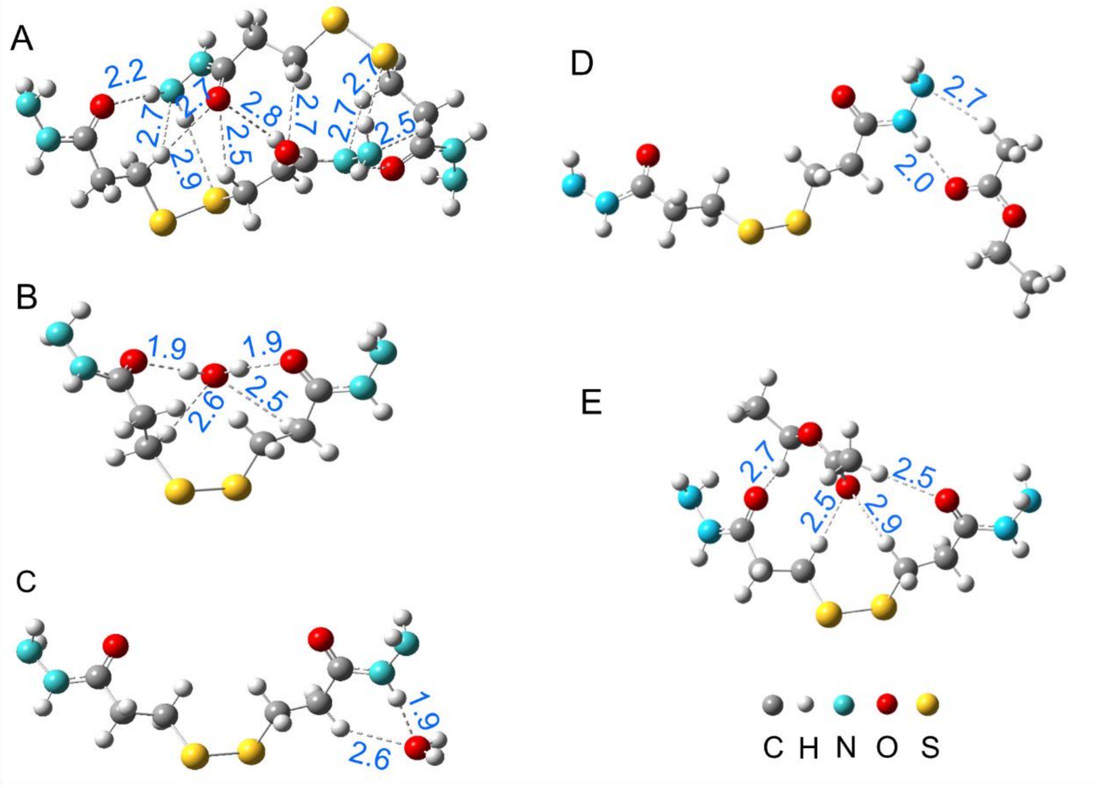

Figure S19. DFT calculation of H-bonding. DFT calculated intermolecular H-bonds between DTPH molecules themselves (A,B), C=O···H-O-H and N-H···O-H typed H-bonds between DTPH and water molecules (C,D), and H-bonds between DTPH and urea molecules (E). Bond lengths are reported in Å.

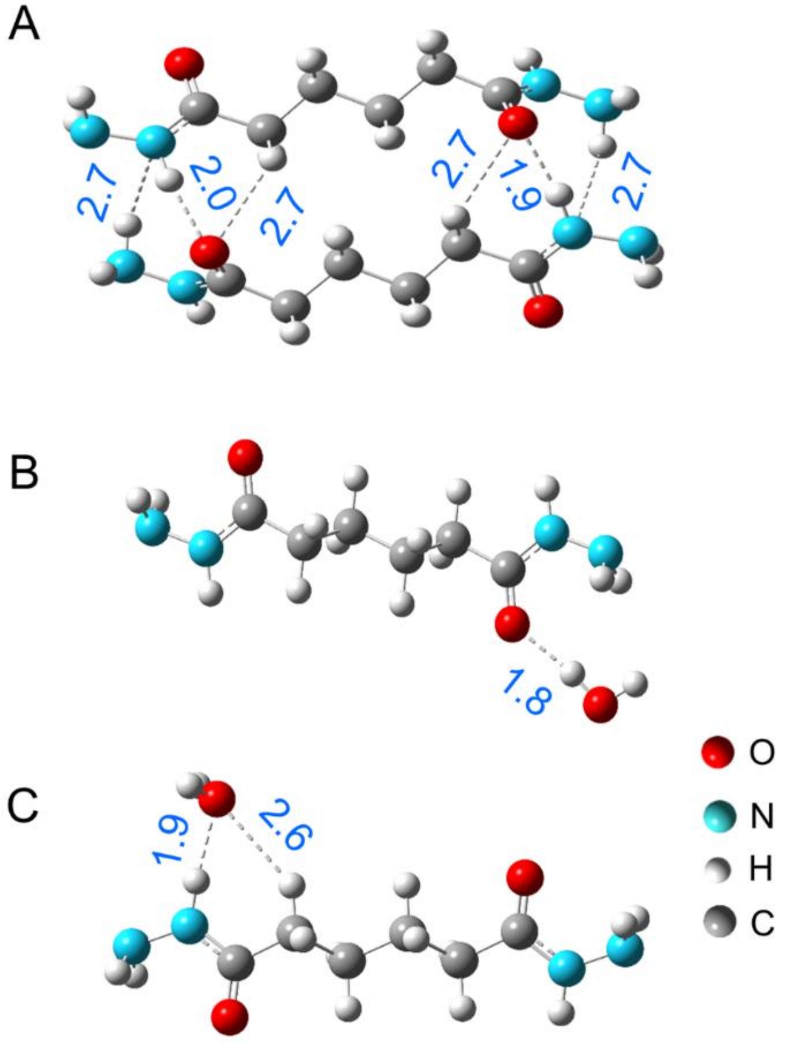

Figure S20. DFT calculation of H-bonding. DFT calculated intermolecular H-bonds between ADH molecules themselves (A) and C=O···H-O-H and N-H···O-H typed H-bonds between ADH and water molecules (B,C). Bond lengths are reported in Å.

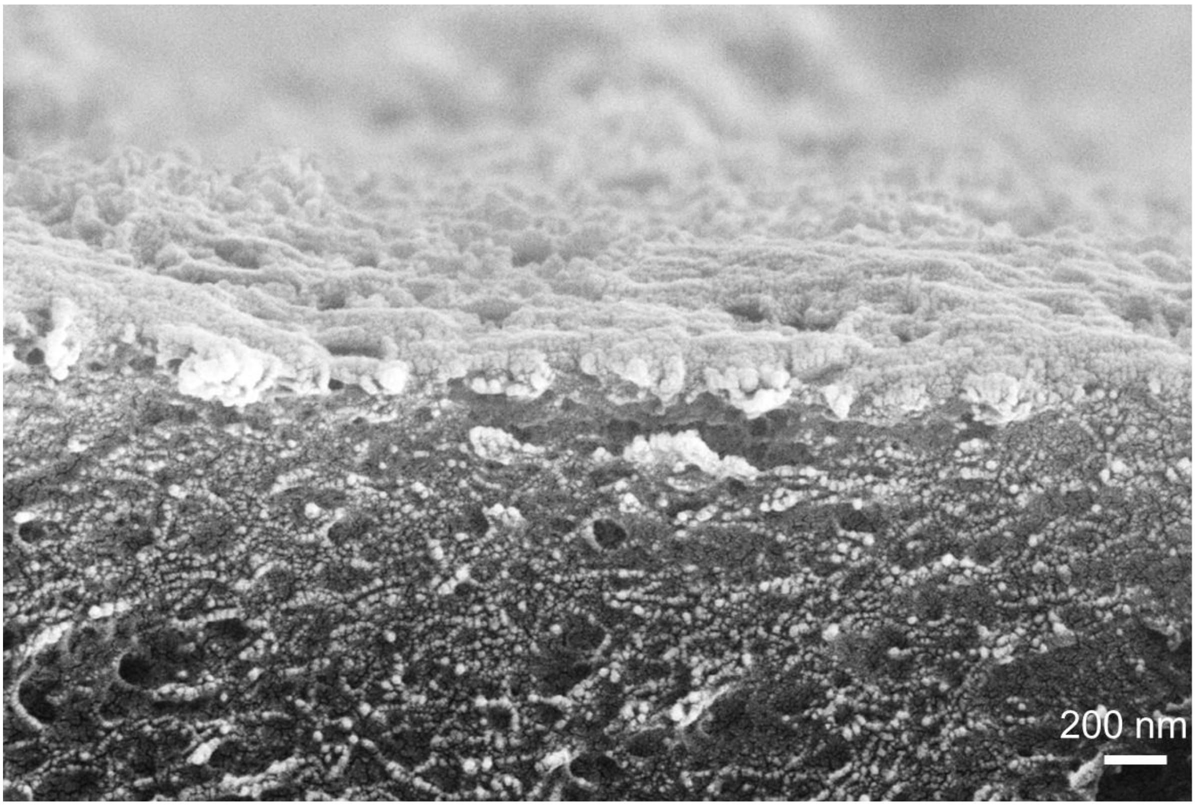

Figure S21. SEM characterization of membrane obtained from the assembly of ADH and BTA. SEM image of the membrane formed by the self-assembly of adipic dihydrazide (ADH) and BTA at the EtOAc/water interface.

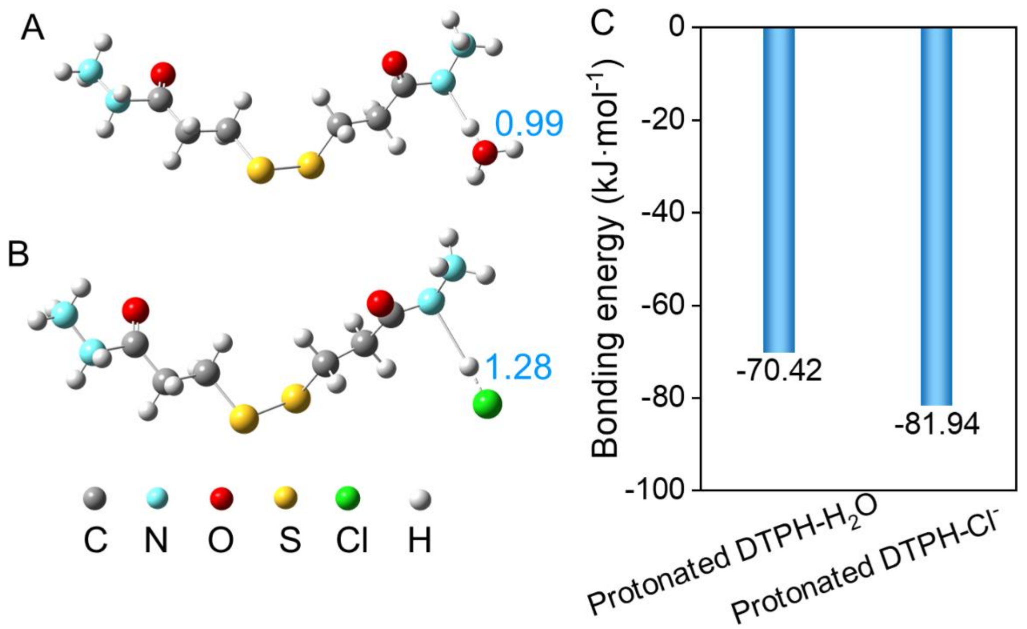

Figure S22. DFT calculated intermolecular H-bonds between protonated DTPH and H_2O (A) and between protonated DTPH and Cl^- (B) and the corresponding H-bonding energies (C). Bond lengths are reported in Å.

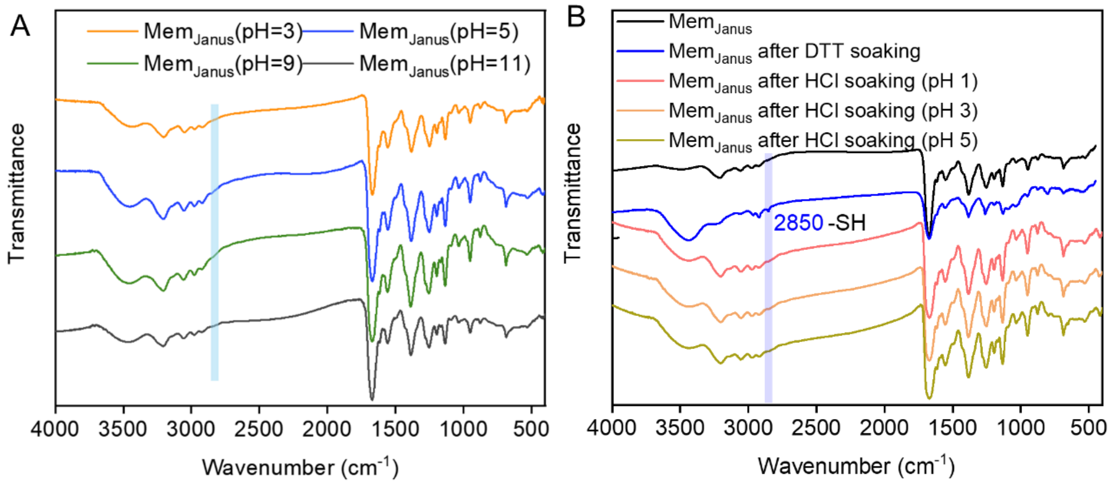

Figure S23. FT-IR spectra of Mem_{Janus} prepared at different pH (i.e., pH = 3, 5, 9, 11) (A) and Mem_{Janus} after DTT soaking and HCl soaking at different pH (i.e., pH =1, 3, 5).

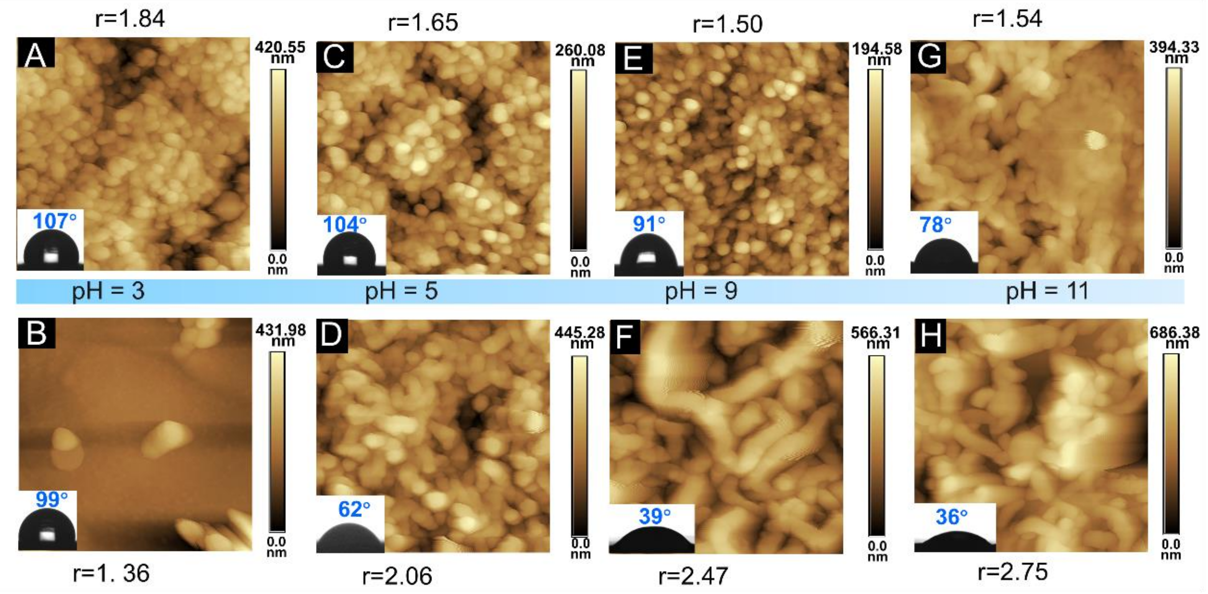

Figure S24. Surface morphology and roughness detected via AFM. AFM images and the roughness factor (r) of Layer-Et (upper) and Layer-Aq (down) of the membrane via the self-assembly of BTA and DTPH at different pH. (A,B) pH = 3; (C,D) pH = 5; (E,F) pH = 9; (G,H) pH = 11. r is the surface roughness factor, which is also called as the roughness ratio, the ratio of actual area and projection area of the surface. Projection area: 2um *2um.

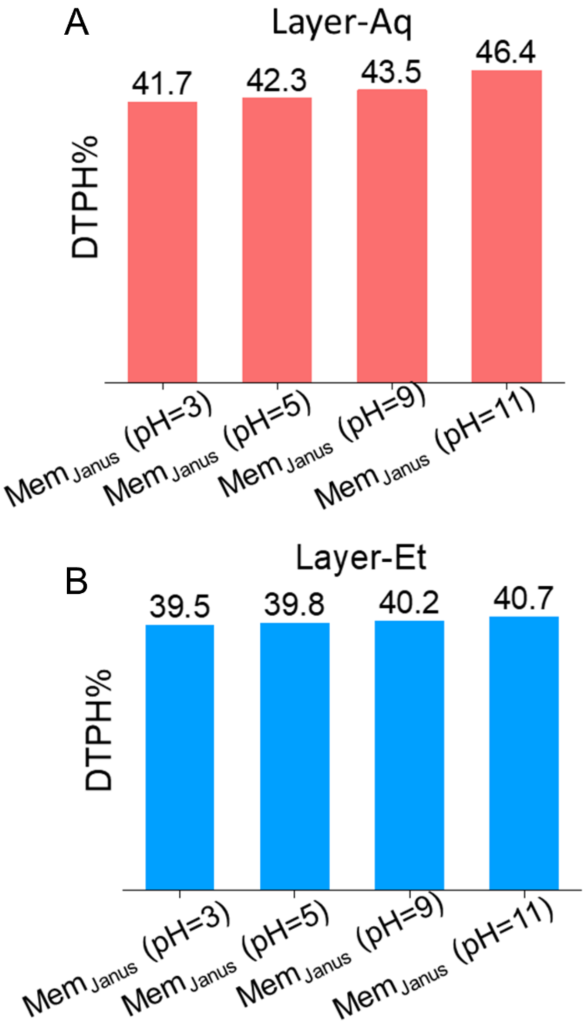

Figure S25. Molar content of DTPH on the opposite sides of the Janus membranes prepared at different pH conditions. (A) Layer-Aq surface; (B) Layer-Et surface.

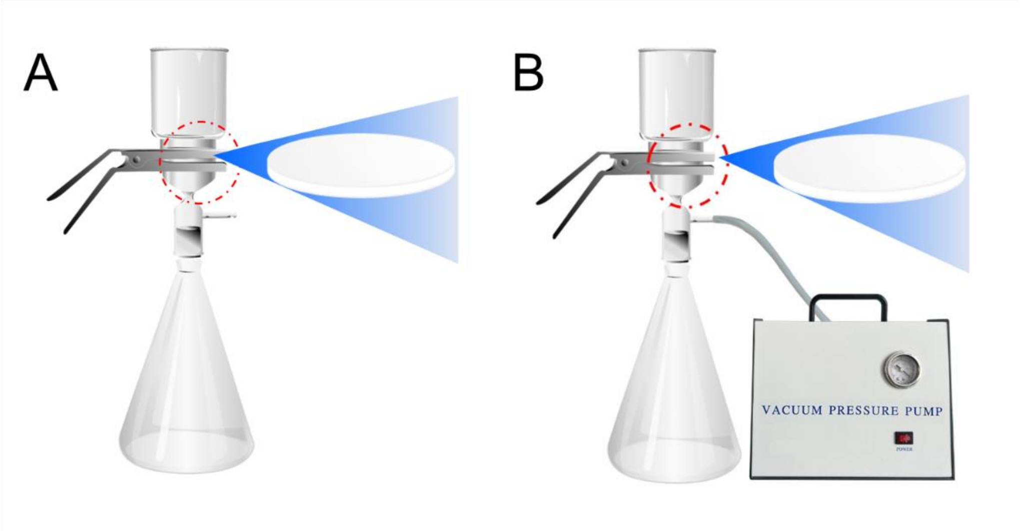

Figure S26. Schematic diagram of water transport devices. Test conditions: natural gravity (A) and vacuum pressure (0.4 bar) (B).

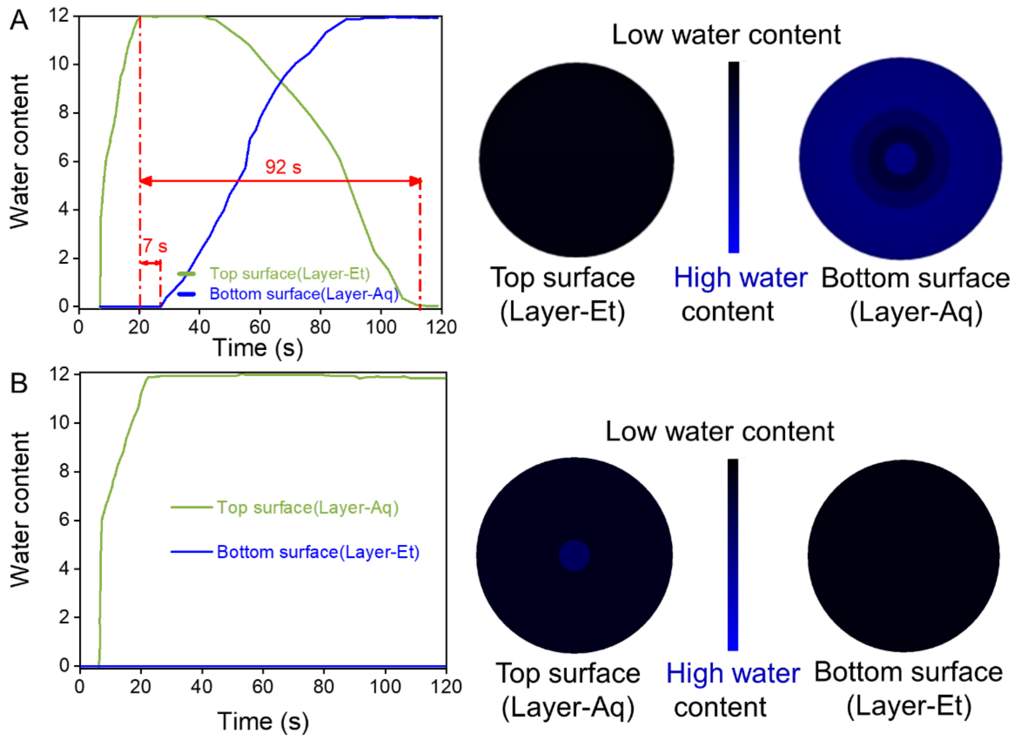

Figure S27. Moisture management profiles plotted against time from Layer-Et to Layer-Aq (A) and from Layer-Et to Layer-Aq (B). The blue region and black region in the photos symbolize high water level and low water level, respectively. The membrane was formed through the assembly of BTA (0.05 M) and DTPH (0.075M) at the EtOAc/water interface at 40 °C for 24h.

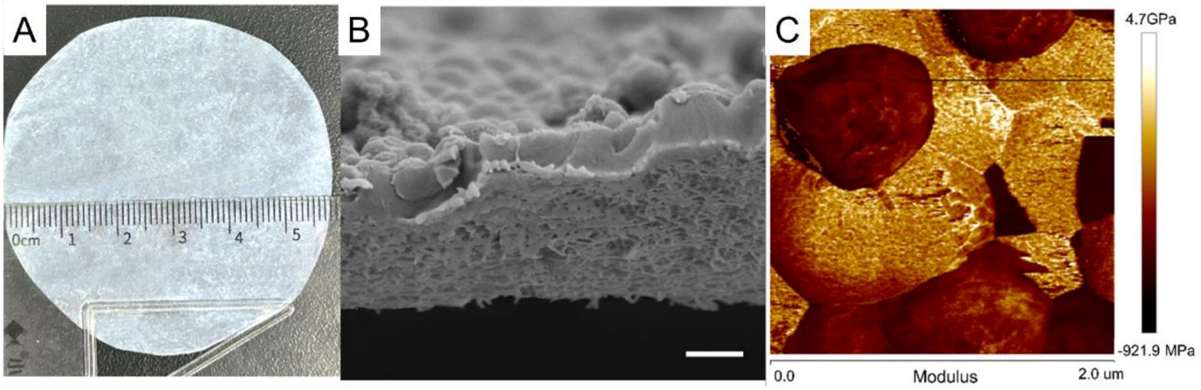

Figure S28. Characterization of used membrane (prepared at pH = 9) after water flux detection. (A) Photograph, (B) SEM image and (C) Young's modulus detected from AFM. Scale bar in SEM: 1um. The membrane was dried under 40 °C after water flux detection under negative-pressure condition (0.4 bar).

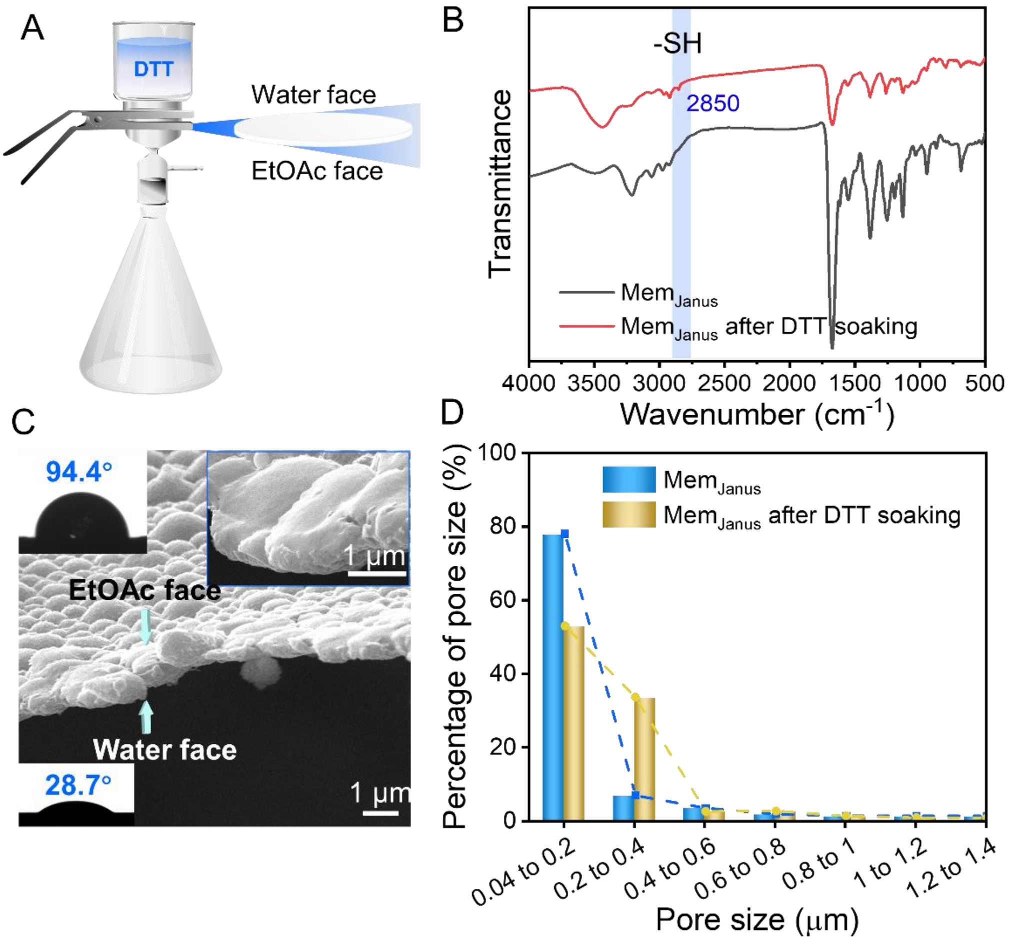

Figure S29. Structural characterizations of the Janus membrane after DTT soaking. (A) Schematic diagram of the device for soaking the hydrophilic layer of the Janus membrane in 1 M aqueous solution of dithiothreitol (DTT) for 2 h. (B) FT-IR spectra of the Janus membrane before and after DTT soaking. (C) SEM image of the Janus membrane after DTT soaking. (D) Pore size distributions of the Janus membrane before and after DTT soaking. The Janus membrane was formed via the interfacial self-assembly of BTA (0.05 M) and DTPH (0.075 M) at the EtOAc-water interface at 40 °C.

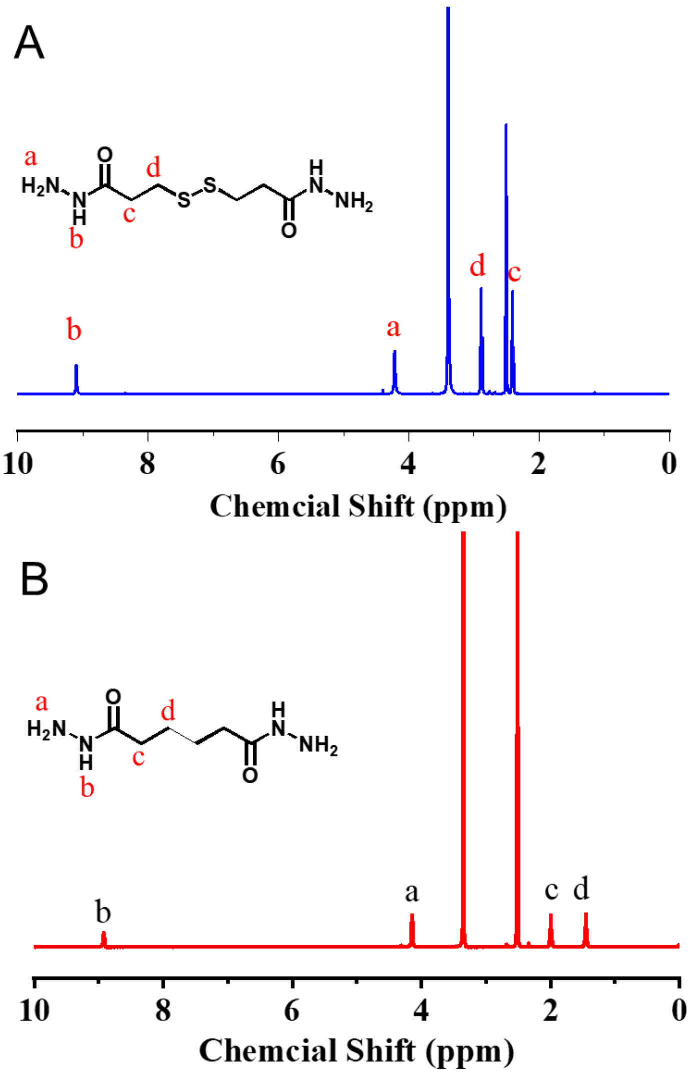

Figure S30. Structural characterizations of building blocks. ^{1}H NMR spectra of 3,3' – dithiobis (propionyl hydrazine) (**A**) and adipic dihydrazide (**B**).

3. Supplementary Tables:

Table S1 Young's modulus of some membrane materials

Materials	Modulus (GPa)	Method	Ref
SWCNT film	5	Tensile	[10]
HPCNT-6	3.461	Tensile	[11]
Superhydrophobic polyvinylidene fluoride (PVDF) membranes	0.07129	Tensile	[12]
pGAA2	0.217	Tensile	[13]
$Poly_{3/6}COF$-42	0.91	Tensile	[14]
COF-505	12.5	AFM Nanoindentation	[15]
Amyloid fibrils	3.2	PF-QNM	[16]
ZIF-8 MOF-CVD	3.44	Nanoindentation	[17]
Mem_{Janus}	1.8	AFM	This work
	1.1	Tensile	

Table S2 Molar content of elements and DTPH on the opposite sides of the Janus membranes prepared at different pH conditions.

Sample[a)]		Molar content of elements				Molar content of DTPH (%)[b)]
		C	N	O	S	
Mem_{Janus} (pH=3)	Layer-Et	68.6	12.0	12.5	6.9	39.5
	Layer-Aq	67.5	12.4	12.9	7.3	41.7
Mem_{Janus} (pH=5)	Layer-Et	69.3	12.1	11.5	7.1	39.8
	Layer-Aq	68.0	12.8	11.8	7.4	42.3
Mem_{Janus} (pH=9)	Layer-Et	67.3	12.3	13.5	7.0	40.2
	Layer-Aq	67.6	13.5	11.2	7.7	43.5
Mem_{Janus} (pH=11)	Layer-Et	67.4	11.7	13.8	7.1	40.7
	Layer-Aq	67.0	13.6	11.3	8.2	46.4

[a)] Membranes formed by the self-assembly of BTA (0.05 M) and DTPH (0.075 M) at EtOAc/water interface for 24 h. [b)] Calculated according to the following equations: $Number\ of DTPH = \frac{A_S*100\%}{2}$; $A_{C(DTPH)} = \frac{A_S}{2} * 6$; $Number\ of\ BTA = \frac{(A_C - A_{C(DTPH)})*100\%}{9}$; $Content\ of\ DTPH(\%) = \frac{number\ of\ DTPH}{number\ of\ BTA + number\ of\ DTPH} * 100\%$, where A_S and A_C is the atomic percent of S and C obtained from XPS, respectively. 2 and 6 is the number of S and C in a DTPH molecule, and 9 is the number of C in a BTA molecule, respectively.

Table S3 Water flux performances of typical reported membranes.

Entry	Sample	Operation method	Pressure (bar)	Water flux ($L·m^{-2} h^{-1} bar^{-1}$)	Ref
1	UiO-66-$NH_{2(1)}$ @PAA	dead-end stirred filtration	0.1	2330	[18]
2	Membrane#3	dead-end cell	1	10384±519	[19]
3	CNCs-coated	dead-end vacuum filtration	vacuum	≥ 9000	[20]
4	PVDF/Fe-TA-SP_{422}	dead-end filtration device	0.4	8699.3	[21]
5	PWP	Negative pressure vacuum filtration	-	15860	[22]
6	CNT@CS/TA-FeOOH	vacuum filtration	0.5	8200	[23]
7	$TAMoS_2$	vacuum filtration	0.7	15,000 ± 100	[24]
8	SWCNT/PD/PEI	vacuum filtration	0.5	7270	[25]
9	CNT wall membrane	dead-end membrane filtration	1 (N_2)	29,600±460	[26]
10	CNT/TiO2	vacuum filtration	0.2	40000	[27]

Table S4 MD Simulated systems.

System	Sys. number	Solvent	Number of PBD	Final box size (nm×nm×nm)
PBD in bulk solvent	1	EtOAc	72	14.9×14.9×14.9
	2	Toluene	72	14.8×14.8×14.8
	3	Hexane	72	14.9×14.9×14.9
	4	Water	72	15.1×15.1×15.1
PBD at solvent/water interface	5	EtOAc	72	10.8×10.8×25.4
	6	Toluene	72	10.8×10.8×25.3
	7	Hexane	72	10.9×10.9×25.5
Pure solvent/water interface	8	EtOAc	0	10.5×10.5×20.3
	9	Toluene	0	10.5×10.5×21.4
	10	Hexane	0	10.5×10.5×20.2

Table S5 MD simulated number of H-bonds between different components at different systems.

Sys.[a)]	Solvent	Water-solvent	Water-PBD	PBD-Solvent	PBD-PBD
1	EtOAc	-	-	92	240
2	Toluene	-	-	0	271
3	Hexane	-	-	0	276
4	Water	-	-	646	163
5	EtOAc	430	353	55	202
6	Toluene	0	541	0	179
7	Hexane	0	606	0	192
8	EtOAc	478	-	-	-
9	Toluene	0	-	-	-
10	Hexane	0	-	-	-

[a)]Sys. 1-4: PBD molecules located in EtOAc (sys. 1), toluene (sys. 2), hexane (sys. 3) and water (sys. 4); Sys. 5-7: PBD molecules located at the EtOAc/water interface (sys. 5), at the toluene/water interface (sys. 6) and at the hexane/water interface (sys. 7); Sys. 8-10: EtOAc/water interface (sys. 8), toluene/water interface (sys. 9), hexane/water interface (sys. 10).

Table S6 MD simulated thickness of interfacial region.

Solvent	Thickness (nm) of interfacial region	
	pure	with PBD
EtOAc	1.3	3.8
Toluene	1.1	2.3
Hexane	0.8	1.9

Table S7 MD simulated radius of gyration (R_g), principal radii of gyration (R_x, R_y, R_z), and solvent accessible surface area (SASA) for PBD aggregates in bulk solvents.[a]

Solvent	R_g (nm) [a)]	R_x (nm) [a)]	R_y (nm) [a)]	R_z (nm) [a)]	SASA (nm^2)[a)]
EtOAc	3.09±0.02	2.00 ±0.01	2.67 ±0.02	2.83 ±0.02	516.68±8.00
Toluene	2.98 ±0.01	2.06 ±0.01	2.45±0.01	2.74 ±0.01	402.71±4.02
Hexane	2.83 ±0.01	2.00±0.01	2.33±0.01	2.57±0.01	305.29±5.55
Water	2.73±0.01	1.83 ±0.01	2.23 ±0.01	2.47 ±0.01	262.64±3.29
Water/EtOAc (2:98 vol.)	3.14±0.03	2.15±0.02	2.60±0.04	2.88±0.03	489.92±6.71
Water/EtOAc (90:10 vol.)	3.25±0.03	2.08±0.02	2.88±0.03	2.90±0.03	502.32±8.38

[a)] Values are the average (with standard deviation) over the last 10 ns of simulations.

References

[1] M. J. Frisch, *Gaussian 16 Rev. C.01*, Gaussian, Inc., Wallingford CT, **2016**.

[2] A. D. Becke, *J. Chem. Phys*. **1993**, *98*, 1372‑1377.

[3] C. M. Breneman, K. B. Wiberg, *J. Comput. Chem*. **1990**, *11*, 361–373.

[4] C. Jian, T. Tang, *J. Phys. Chem. B* **2014**, *118*, 12772–12780.

[5] Dortmund Data Bank, **2022**, www.ddbst.com.

[6] M. O. McLinden, J. D. Splett, *J. Res. Natl. Inst. Stand. Technol.* **2008**, *113*, 29.

[7] T. Lan, H. Zeng, T. Tang, *J. Phys. Chem. C* **2018**, *122*, 28787–28796.

[8] H. N. Banavath, O. P. Sharma, M. S. Kumar, R. Baskaran, *Sci Rep*. **2014**, *4*, 6948.

[9] Y. Y. Zhou, K. A. Gammeltoft, L. A. Ryberg, L. V Pham, H. D. Tjørnelund, A. Binderup, C. R. D. Hernandez, C. Fernandez-Antunez, A. Offersgaard, U. Fahnøe, G. H. J. Peters, S. Ramirez, J. Bukh, J. M. Gottwein, *Sci. Adv*. **2022**, *8*, eadd7197.

[10] X. F. Zhang, T. V. Sreekumar, T. Liu, S. Kumar, *J. Phys. Chem. B* **2004**;*108*, 16435–40.

[11] Q. Duan, S. Y. Wang, Q. F. Wang, T. Li, S. F. Chen, M. H. Miao, D. H. Zhang, *ACS Appl. Mater. Interfaces* **2019**, *11*, 36278−36285.

[12] K. J. Lu, C. Z. Liang, Y. L. Chen, T. S. Chung, *Desalination* **2022**,*527*, 115579.

[13] Y. Kim, J. Kim, C. Y. Kim, T. Kim, C. Lee, K. Jeong, W. Jo, S. Yoo, T. Kim, K. C. Choi, S. G. Im, Chem. Eng. J. **2022**, *431*, 134074.

[14] Z. F. Wang, Q. Yu, Y. B. Huang, H. D. An, Y. Zhao, Y. F. Feng, X. Li, X. L. Shi, J. J. Liang, F. S. Pan, P. Cheng, Y. Chen, S. Q. Ma, Z. J. Zhang, *ACS Central Sci*. **2019**, *5*, 1352–1359.

[15] Q. Hao, C. Q. Zhao, B. Sun, C. Lu, J. Liu, M. J. Liu, L. J. Wan, D. Wang, *J. Am. Chem. Soc*. **2018**, *140*, 12152–12158.

[16] J. Adamcik, C. Lara, I. Usov, J. S. Jeong, F. S. Ruggeri, G. Dietler, H. A. Lashuel, I. W. Hamley, R. Mezzenga, *Nanoscale* **2012**, *4*, 4426–4429.

[17] M. Krishtab, I. Stassen, T. Stassin, A. J. Cruz, O. O. Okudur, S. Armini, C. Wilson, S. D. Gendt, R. Ameloot, *Nat. Commun*. **2019**, *10*, 3729.

[18] J. Cao, Y. Su, Y. Li, J. Guan, M. He, R. Zhang, Z. Jiang, *J. Membrane Sci*. **2018**, *566*, 268–277.

[19] Y. Zhou, J. Zhang, Z. Wang, F. He, S. Peng, Y. Li, *J. Membrane Sci*. **2021**, *618*, 118703.

[20] J. Zhang, M. Pan, J. Zhang, L. Zhang, F. Lin, X. Liu, C. Huang, X. Z. Chen, J. Wang, B. Yan, H. Zeng, *Adv. Funct. Mater*. **2021**, *32*, 2109989.

[21] H. Xie, L. Shen, Y. Xu, H. Hong, L. Yang, R. Li, H. Lin, *J. Membrane Sci*. **2022**, *660*, 120873.

[22] Z. Huang, L. Shen, H.Lin, B. Li, C. Chen, Y. Xu, R. Li, M. Zhang, D. Zhao, *J. Membrane Sci*. **2022**, *661*, 120949.

[23] X. Zhao, L. Cheng, N. Jia, R. Wang, L. Liu, C. Gao, *J. Membrane Sci*. **2020**, *600*, 117857.

[24] W. Hu, X. Cui, L. Xiang, L. Gong, L. Zhang, M. Gao, W. Wang, J. Zhang, F. Liu, B. Yan, *J. Colloid. Interf. Sci*. **2020**, *560*, 177-185.

[25] S. J. Gao, Y. Z. Zhu, F. Zhang, J. Jin, *J. Mater. Chem. A* **2015**, *3*, 2895-2902.

[26] B. Lee, Y. Baek, M. Lee, D. H. Jeong, H. H. Lee, J. Yoon, Y. H. Kim, *Nat. Commun*. **2015**, *6*, 7109.

[27] L. Yan, C. Liu, J. Xia, M. Chao, W. Wang, J. Gu, T. Chen, *J. Clean. Prod*. **2020**, *275*, 124011.
